# Functional Ink Formulation for Printing and Coating of Graphene and Other 2D Materials: Challenges and Solutions

**DOI:** 10.1002/smsc.202200040

**Published:** 2022-10-02

**Authors:** Mohammad Jafarpour, Frank Nüesch, Jakob Heier, Sina Abdolhosseinzadeh

**Affiliations:** ^1^ Laboratory for Functional Polymers Swiss Federal Laboratories for Materials Science and Technology (Empa) 8600 Dübendorf Switzerland; ^2^ Institute of Materials Science and Engineering Swiss Federal Institute of Technology Lausanne (EPFL) 1015 Lausanne Switzerland

**Keywords:** 2D materials, exfoliation, functional inks, graphene, printing

## Abstract

The properties of 2D materials are unparalleled when compared to their 3D counterparts; many of these properties are a consequence of their size reduction to only a couple of atomic layers. Metallic, semiconducting, and insulating types can be found and form a platform for a new generation of devices. Among the possible methods to utilize 2D materials, functional printing has emerged as a strong contender because inks can be directly formulated from dispersions obtained by liquid‐phase exfoliation. Printed graphene‐based devices are shifting from laboratory applications toward real‐world and mass‐producible systems going hand in hand with a good understanding of suitable exfoliation methods for the targeted type of ink. Such a clear picture does not yet exist for hexagonal boron nitride (h‐BN), the transition metal dichalcogenides (TMDs), and black phosphorous (BP). Rather, reports of applications of these 2D materials in printed devices are scattered throughout the literature, not yet adding to a comprehensive and full understanding of the relevant parameters. This perspective starts with a summary of the most important features of inks from exfoliated graphene. For h‐BN, the TMDs, and BP, the characteristic properties when exfoliated from solution and strategies to formulate inks are summarized.

## Graphical abstract

2D materials are promising candidates for printing electronics, but their commercial applications still face numerous challenges. Unlike nonfunctional printing where additives are readily used to address the printing challenges, in the functional printing of 2D materials, it is necessary to seek other, innovative solutions and optimize all stages of the device fabrication, from exfoliation to ink formulation, and printing.

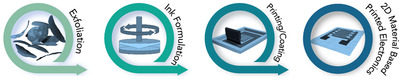

## 1 Introduction

In recent years, 2D materials such as graphene, black phosphorus (BP), hexagonal boron nitride (h‐BN), and transition metal dichalcogenides (TMDs) with their diverse and exceptional properties have attracted enormous attention in various fields of science and technology, from electronics to sensing, catalysis, and energy storage/conversion. There are several methods for integrating 2D materials in different applications, among which solution processing as a simple, scalable, and cost‐efficient material processing method is arguably the most widely used and studied technique. This is because most 2D materials can be produced either via liquid‐phase exfoliation (LPE) of their parent layered crystals (usually naturally abundant) or solution‐based synthesis routes.^[^ ^1^ ^]^ As a result, highly scalable and industrial manufacturing techniques such as printing and wet coating can be used for their incorporation in commercial applications and device fabrication.^[^ ^2^ ^]^

Despite the significant advancements in the past two decades, the processing of 2D materials into functional inks and their efficient printing and coating still face numerous challenges. The low yield of the exfoliation processes and the difficulty of producing stable high‐concentration dispersions of 2D materials are two of the main obstacles to the formulation of functional inks. Low concentration inks necessitate multiple overlayer printings,^[^ ^3^ ^]^ compromising the print resolution and prolonging the manufacturing time. Exfoliation processes usually yield products with a broad particle size distribution.^[^ ^4^ ^]^ The lateral size of the flakes, the number of layers in each flake, and their aspect ratio strongly determine the electrical properties of the films^[^ ^5^ ^]^ (number of the intersheet junctions and bandgap in semiconductor 2D materials), the flow behavior of the inks, and their printability using different printing methods.^[^ ^6^ ^]^ Therefore, controlling the 2D nanosheet morphological parameters is very important for obtaining the best performance and reproducible results. The low compaction level (high porosity) of the printed/coated 2D nanosheet‐based films is another issue^[^ ^7^ ^]^ that should be considered when fabricating devices using printing and coating methods.

Fulfilling the rheological requirements of the different printing methods (e.g., high viscosity inks for screen‐ and extrusion‐printing) is another challenge that limits the application of low‐concentration inks to a few methods such as inkjet printing (IJP) and aerosol jet printing (AJP), which rely on low‐viscosity inks.^[^ ^8^ ^]^ The surface tension of the dispersing solvents (which determines the wettability and adhesion of the printed films) and their chemical compatibility (with substrate and other components of the device) are two different issues that make the functional ink formulation even more difficult.^[^ ^6^ ^]^ Several attempts have been made to address these challenges, most of which are inspired by the conventional techniques used in nonfunctional printing and coating.^[^ ^9^ ^]^ Except for a few instances, these traditional solutions are usually not suitable for functional printing, necessitating the development of innovative approaches. In this contribution, some of the most important works on printing and wet coating of 2D materials are reviewed, and the most effective solutions for the aforementioned challenges are discussed. Since all common components of an electronic circuit (both active and passive) are composed of either conductor, semiconductor, or insulator materials, and considering the wide range of electronic properties that 2D materials can offer, it is possible to realize fully printed electronics by printing and coating of 2D materials. In this respect, the reviewed 2D materials are classified and ordered based on their electronic properties. Basics of the solution processing, the exfoliation/synthesis of 2D materials and the introduction of different printing and coating methods have been extensively reviewed elsewhere^[^ 8, 10 ^]^ and will not be covered here in detail. We hope this perspective will serve as a guideline for those who intend to integrate 2D materials in their applications using printing and wet coating methods.

## 2 Conductive Inks

### 2.1 Graphene

#### 2.1.1 Exfoliation and Preprocessing

Graphene, the single layer of sp^2^‐bonded carbon atoms, is the most extensively studied member of the 2D materials family. Pristine graphene is an excellent electrical and thermal conductor, and its properties can be easily tuned via chemical functionalization or doping.^[^ ^11^ ^]^ Since its parent crystal is cheap and naturally abundant, LPE is the most preferred technique for its commercial production.^[^ ^2^ ^]^ In general, graphite's most widely used exfoliation techniques can be classified into two groups: chemical and mechanical methods.

In the chemical methods, the main goal is to weaken the van der Waals attractions between the graphene layers in the graphite's crystal by increasing the interlayer distances either by adding functional groups to the graphene layers or intercalating chemical species to their interlayer galleries.^[^ ^12^ ^]^ These methods offer very high exfoliation yields and are usually used for the commercial production of “graphene derivatives”. The term “derivative” is used here since the graphene products obtained via these routes are usually chemically functionalized to a different degree, depending on the exfoliation method. While the functionalization can improve the graphene's solution processability and performance in some applications (e.g., sensing, energy storage), it severely affects its electronic properties and may not be desirable in some other applications (e.g., when it is used as a current conductor). Considering the vast diversity of the functional groups of graphene and the huge number of techniques that have been developed for the synthesis and processing of the functionalized derivatives of graphene, it is not possible to include such materials (e.g., graphene oxide and reduced graphene oxide) in this review as well.

In the mechanical exfoliation methods, pristine graphene is produced by applying shear forces to graphite powder in organic solvents (or solvent mixture) with matching surface energy such as *N*‐methyl‐2‐pyrrolidone (NMP), which minimizes the energetic cost of the exfoliation process. Unfortunately, the exfoliation yield of the mechanical methods and consequently the concentration of the resultant dispersions are often considerably lower than their chemical counterparts. Although by increasing the exfoliation time, and the power of the applied mechanical forces, the concentration of the dispersions can be increased, the obtained 2D nanosheets are usually more defective, and have smaller flake sizes,^[^ ^13^ ^]^ which yields films with inferior electrical properties. While its origin is not clearly known,^[^ ^14^ ^]^ it has been shown by zeta potential measurements that the pristine graphene (unfunctionalized) possesses surface charges (both positive and negative, depending on the solvent), which can be the main reason for the stability of the obtained graphene dispersions.^[^ ^15^ ^]^ Based on a correlation between the donor number of the dispersion solvent and the sign of the zeta potential for the dispersed graphene nanosheets, it has been suggested that the surface charge is a result of electron transfer between the graphene and the dispersing solvent.^[^ ^15^ ^]^ Dissociation of oxygen‐containing functional groups, which are usually present in the starting raw graphite (as impurities or in defect sites/edges), may also contribute to the charging of nonaqueous graphene dispersions.^[^ ^14^ ^]^

The limited dispersibility of pristine 2D materials in pure solvents is mainly due to the electrostatic stabilization of their dispersion, where increasing the concentration above a certain threshold would lead to the destabilization of the suspension.^[^ ^16^ ^]^ Suspensions with higher concentrations can be obtained by stabilizing the graphene nanosheets using surfactants and polymers. However, the maximum concentration can hardly reach 1–2 mg mL^−1^, which may be acceptable for some deposition techniques (e.g., spray coating or IJP) but is still very low for most other printing methods. Furthermore, as will be discussed later, addition of surfactants and polymers can affect the electronic properties of the printed materials and should be avoided wherever possible.

**Figure 1:**
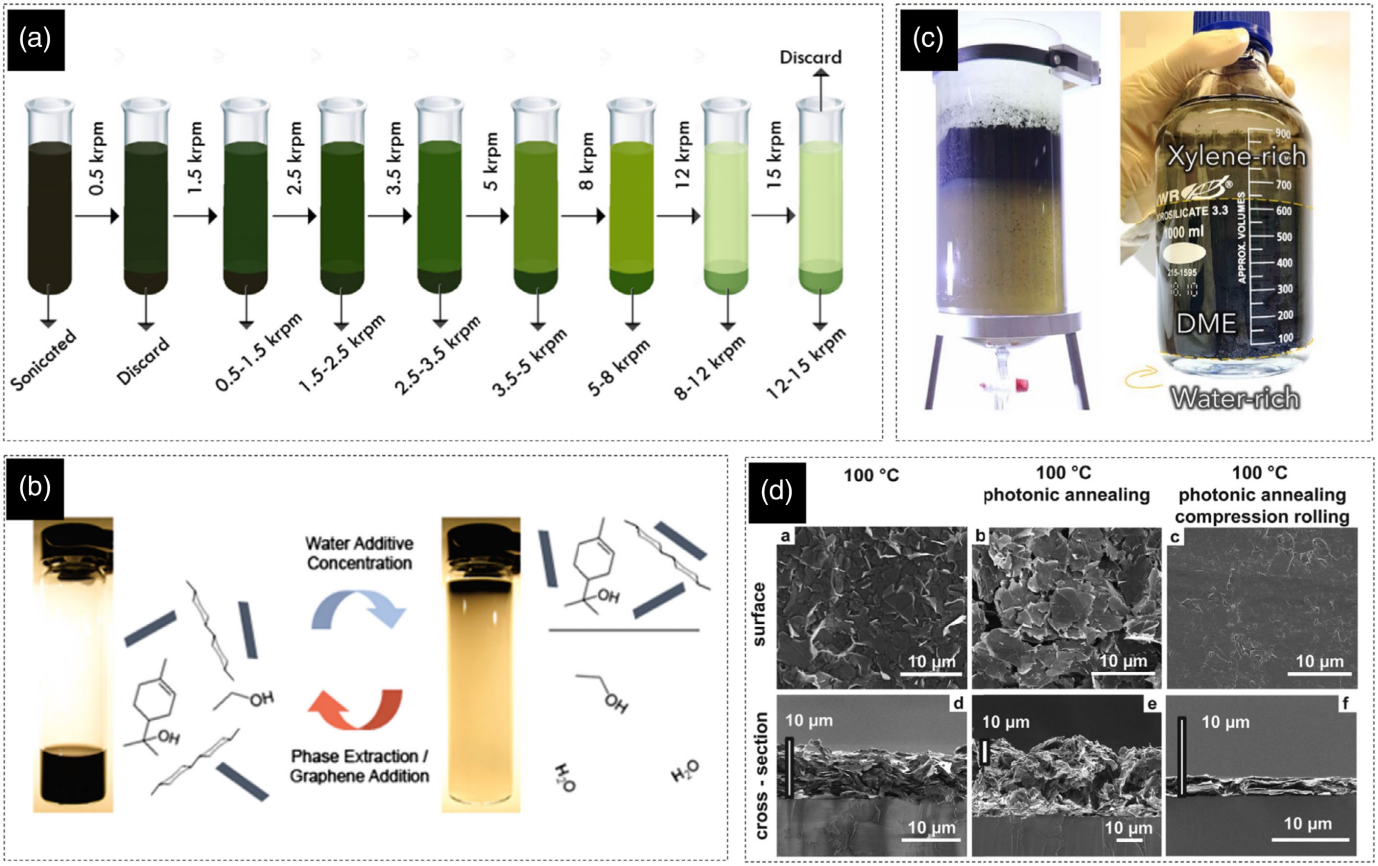
a) Schematic demonstration of the cascade centrifugation method. Reproduced with permission.^[^ ^17^ ^]^ Copyright 2016, American Chemical Society. b) Separation of graphene/ethyl cellulose from stable dispersion by addition of water. Reproduced with permission.^[^ ^18^ ^]^ Copyright 2010, American Chemical Society. c) Interface‐assisted extraction of graphene from NMP by the formation of a Pickering emulsion. Reproduced under the terms of the CC‐BY Creative Commons Attribution 4.0 International license (https://creativecommons.org/licenses/by/4.0).^[^ ^6^ ^]^ Copyright 2022, The Authors, published by Wiley‐VCH. d) Volumetric changes (compaction level) of the graphene film after photonic annealing and compression rolling. Reproduced with permission.^[^ ^19^ ^]^ Copyright 2016, Wiley‐VCH.

Therefore, to formulate inks for efficient printing and coating, it is often necessary to increase the concentration of the exfoliated 2D nanosheets using additional processes. It is worth noting that since the exfoliation products have a very wide particle size distribution, it is usually beneficial to perform flake size screening after the exfoliation step and before making any attempt to process the dispersion into an ink. This is important from several aspects; first, some printing methods can only handle particles in a specific size range (e.g., inkjet printers); second, the rheological properties of the inks will be more consistent from one exfoliation batch to another; and third, the performance of the printed devices will be more reproducible and reliable since the electronic properties of the 2D materials heavily depend on the size and the thickness of their flakes (will be discussed later). Size screening can be done by various techniques, most of which are done using centrifugation of the dispersions at different speeds and conditions.^[^ ^10^ ^]^ The simplest yet most practical approach is based on a cascade centrifugation where the suspension is subsequently centrifuged at different speeds (in an increasing order; **Figure** **1**a).^[^ ^17^ ^]^

Separating the exfoliated graphene from the dispersion medium using ultrahigh speed centrifugation and redispersing it in a smaller quantity of solvent is one of the simplest methods for producing high concentration dispersions (increasing the concentration up to 6 mg mL^−1^).^[^ ^3^ ^]^ Despite its simplicity, as this method is based on ultrahigh‐speed centrifugation, the production time is very long, and scaling up the process is challenging. Solvent exchange is another widely used technique in which graphene is first exfoliated in a low boiling point solvent and then transferred to a smaller amount of another solvent with a higher boiling point (by evaporation).^[^ ^20^ ^]^ Similarly, since evaporation is a time‐ and energy‐consuming process, this method is also suitable mainly for lab‐scale investigations.

Considering the high stability of the graphene dispersions, separating the graphene from the dispersion medium is not easily possible. Phase‐separation‐based methods can be used to facilitate this process. The earliest works on this approach are based on dispersing the graphene in an organic solvent (e.g., ethanol) using ethyl cellulose (EC) and then flocculating the obtained dispersions. Flocculation can be initiated using different methods; for instance, by first adding terpineol to the graphene/EC/ethanol dispersion and then adding water to form a hydrophilic ethanol solution and pushing the hydrophobic EC‐covered graphene flakes to terpineol, which is not miscible in water (Figure 1b).^[^ ^18^ ^]^ Another technique for flocculation of the graphene/EC/ethanol dispersion is the addition of a small amount of aqueous sodium chloride solution.^[^ ^21^ ^]^ In both methods, the graphene/EC composite is collected and dried after some purification steps. The obtained dried powder can be readily redispersed in a wide range of solvents with desired concentrations, addressing both the concentration and the surface energy challenges in graphene ink formulation. It should be noted that in addition to the flocculation step, EC plays a vital role in other stages of this process; it first stabilizes the graphene and significantly improves the exfoliation yield, and later protects it from restacking during the flocculation and drying steps. These methods are very fast, efficient, and useful in lots of the cases but cannot be used for applications where the thermal treatment is not possible. EC degrades the electronic properties of graphene and should be removed after printing/coating at high temperature (250–35 °C) thermal treatments.^[^ ^21^ ^]^

Another recently developed phase‐separation‐based technique that is as fast and efficient as the previously discussed methods does not require solid additives and consequently no high‐temperature thermal treatment. In this technique, first, a water‐immiscible solvent such as xylene is added to the graphene dispersion (in organic solvents such as NMP or dimethylformamide (DMF)). Then the phase separation is triggered by the addition of water to the mixture. As a result, graphene is forced to leave the dispersion medium to cover the surface of the xylene microdroplets and minimize the huge surface energy of the emulsion formation. As a result, a pickering emulsion will form in the middle of the three‐phase system (water‐rich phase at the bottom, the pickering emulsion in the middle, and the xylene‐rich phase on top; Figure 1c), which can be easily collected (e.g., using a separation funnel) and used for ink production after some purification steps.^[^ ^6^ ^]^ Pristine graphene can easily restack in water,^[^ ^22^ ^]^ and the formation of the metastable pickering emulsion protects the graphene from restacking during the separation stage. This method can also be used for graphene dispersions with a water‐based medium (e.g., water/ethanol mixture). In this case, by gradually adding a water‐immiscible solvent (e.g., xylene), the pickering emulsion will form after passing the solubility threshold (initially, some amount of the immiscible solvent can be dissolved in the dispersion medium because of the organic solvent).

#### 2.1.2 Ink Formulation and Printing

The ink formulation strategies, their composition, and the specific properties that they should possess to be printable/coatable on various substrates using different printing/coating methods vary considerably. Therefore, the first step before processing a functional material into an ink is the determination of the most suitable deposition technique by considering the restrictions and the requirements of each specific device or application. Some of the most important considerations in this respect are the expected properties that the functional materials should exhibit, the scale or cost at which such devices or films should be produced, the printing resolution, and the geometry of the substrate or device (2D or 3D).

**Figure 2:**
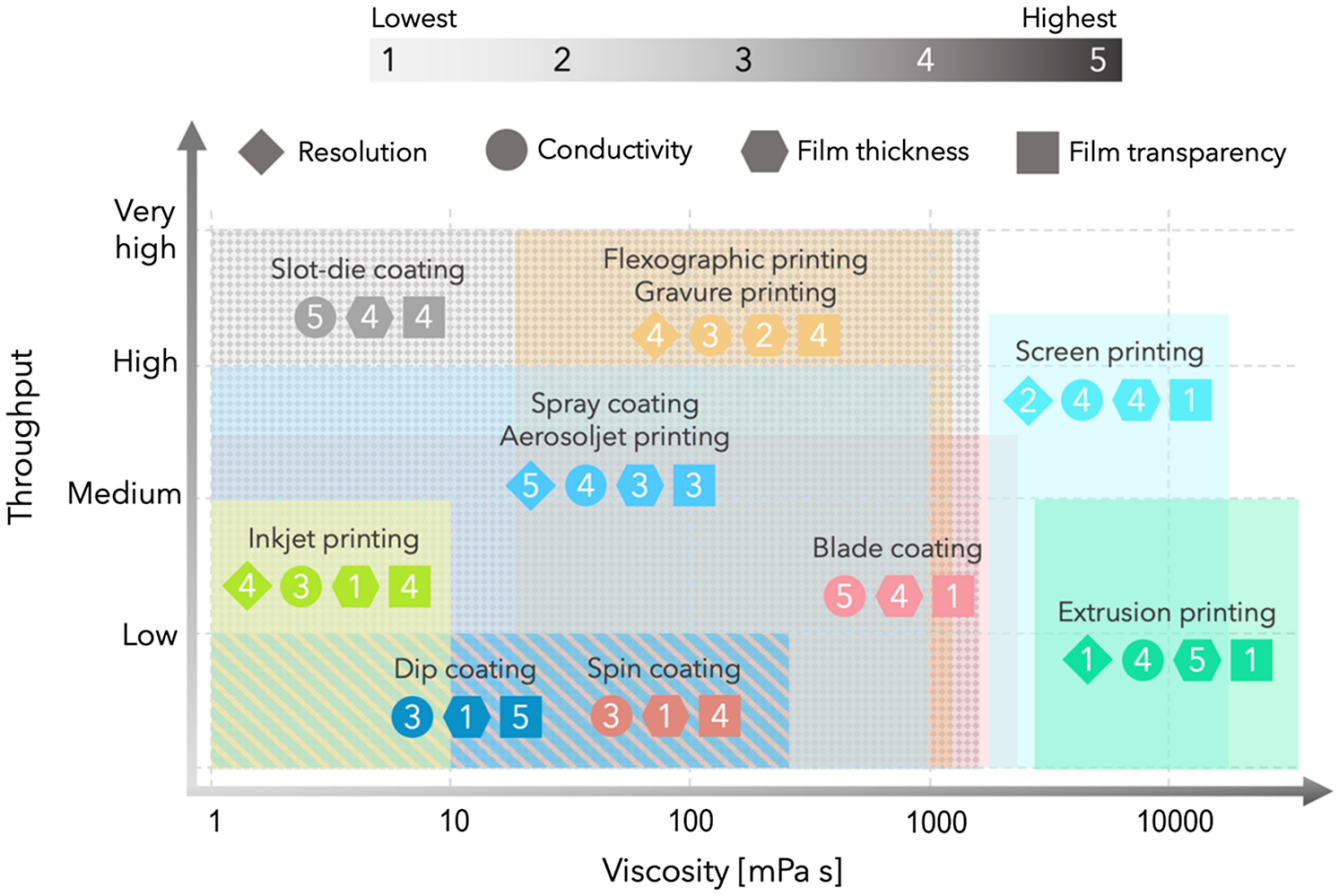
Relation between the ink viscosity and the best achievable throughput, resolution (only applies for printing methods), film conductivity, film thickness, and film transparency for major printing/coating methods. Reproduced with permission.^[^ ^8^ ^]^ Copyright 2021, Elsevier Ltd.

For instance, fabrication of a graphene‐based transparent conductive electrode requires a totally different deposition technique and ink than an interdigitated microsupercapacitor electrode. Since a compact, uniform, and high‐quality thin film (made with large and defect‐free graphene flakes) is required for the transparent electrode, methods such as IJP, aerosoljet printing, spin‐coating, blade coating, and slot‐die coating are potential candidates. However, when considering the expected properties, IJP does not seem to be a good choice as it can only handle small flakes (particle diameter <10% nozzle diameter). Aerosoljet printing also has some limitations on the size of the processable particles (depending on the atomization mechanism), and the alignment and the compaction level of the nanosheets in the deposited films may not be as good as the three other options (spin‐, blade‐, and slot‐die‐coating). Ultimately, three clear choices can be made by considering the production scale; small scale: spin coating, medium to large scale: blade coating, and large‐scale: slot‐die coating. On the other hand, for the fabrication of a microsupercapacitor, usually the deposition of a thick film (with the least number of printing/coating passes) is of greater importance, meaning that methods such as screen‐, and extrusion‐printing are better choices. In **Figure** **2**, which is adapted from reference,^[^ ^8^ ^]^ major determining factors for the selection of the deposition technique for different application and expected properties are summarized. Further information about the working principles of each of the deposition techniques can be found in other dedicated literature.^[^ ^8^ ^]^

Once the most suitable deposition technique is identified, the obtained graphene from the exfoliation step or its “concentrate” can be processed into a printable/coatable ink. The main consideration here is the rheological requirements of that specific deposition method (Figure 2). For instance, inkjet printable inks should possess a viscosity and a surface tension in a certain range to be jettable and to form spherical satellite‐free droplets or screen printable inks should exhibit shear‐thinning behavior and high‐enough viscosity to stay on the mesh openings after flooding the ink and before being transferred to the substrate. While the viscosity is an important characteristic of an ink, optimized printing results can only be obtained when all the rheological properties of an ink such as storage and viscous moduli and thixotropy are fine‐tuned for that specific printing or coating method. Detailed discussions on the rheological requirements of every printing or coating method are far beyond the scope of this perspective.

Wettability (ink on the substrate), film formation behavior (e.g., drying speed, crack formation, and dried film profile), adhesion of the film, cohesion of the particles, thermal stability of the utilized materials (e.g., substrate), and chemical compatibility of the carrier solvents with other components of the device (or substrate) are other important determining factors for ink formulation and selection of its composition. In conventional nonfunctional ink formulations, large quantities of various types of additives such as binders, rheology modifiers, defoamers, and surfactants are readily used for adjusting the properties of the inks and fulfilling these requirements. However, in functional printing, the application of additives is usually minimized since they drastically degrade the electronic properties of the materials. It is worth mentioning here that due to their unique 2D morphology and variable aspect ratio, formulation of 2D‐material‐based inks requires special considerations. This is mainly related to the interactions of the nanosheets in their dispersions, especially when they are single‐ or few‐layered and have high aspect ratio.^[^ ^2^ ^]^ These interactions, which are very considerable even at low concentrations (and become even more substantial at higher concentrations), can both pose certain restrictions for adjusting the ink composition (especially on solid content) and bring up new possibilities for tuning the rheological properties without using additives.

Binders (e.g., macromolecules and polymers) are one of the major components in most of the previously reported graphene inks, especially those that require high viscosities. In these cases, binders act as stabilizing agents for increasing the graphene concentration in the inks and for adjusting their rheological properties.^[^ 23, 24 ^]^ It should be mentioned that the application of the binders is unavoidable in some applications where ultrahigh wear resistance is required or when adhesion to a specific substrate is not easily possible by adjusting the surface tension of the inks.^[^ ^8^ ^]^ Cellulosic binders such as EC and sodium carboxymethyl cellulose are some of the most widely used binders for graphene ink formulation, which require thermal posttreatments between 250 and 35 °C. Enormous efforts have been devoted to developing binders with low decomposition temperature or minimum impact on the electronic properties of graphene.^[^ ^25^ ^]^ For instance, nitrocellulose‐based graphene ink (film) can offer high conductivities (≈10 000 S m^−1^) even when treated at 20 °C. Since decomposition of binders is usually an exothermic reaction (especially nitrocellulose, which also has very fast kinetics), localized and rapid heating methods such as photonic annealing can be used to trigger a self‐propagating combustion reaction that enables the low‐temperature removal of the additives throughout the entire thickness of the printed films.^[^ ^26^ ^]^ It is worth mentioning that despite their detrimental effect on the electronic properties of the functional materials, some cellulosic binders can improve the conductivity of the graphene films after the thermal treatment process because their decomposed moieties that are usually aromatic species with the ability of π–π stacking between the residues and graphene flakes can establish relatively efficient charge transport pathways.^[^ 21, 25 ^]^

Recently, it has been shown that the well‐exfoliated graphene nanosheets can form efficient interlocks and densely packed films (compared to other types of nanomaterials) with acceptable mechanical properties for lots of applications; meaning that in most cases, the addition of a binder to the 2D materials inks is not necessary. It has also been found that a graphene (or any other pristine 2D material) dispersion with high‐enough graphene concentration can be easily processed into a gel. At such high concentrations, the dispersion consists of aggregated particles that can form a continuous 3D network in which the solvents can be dispersed. Since the structure and stability of the dispersion are mainly based on the van der Waals (vdW) interactions of the particles, it is possible to use a wider range of solvents that are even incapable of dispersing the graphene as a suspension medium. As a result, the surface tension of the inks, their wettability, and adhesion of the resultant films to most of the substrates can be easily controlled by changing the solvent type. These structural features also enable the fine‐tuning of the rheological properties just by adjusting the graphene concentration for all printing methods that require high viscosity inks.

Formulation of inks based on capillary suspensions is another creative approach for fulfilling the rheological requirements of the different printing methods without using solid additives. To form a capillary suspension, a small amount of a secondary fluid, immiscible with the continuous phase of the suspension is added, with specific liquid/liquid and liquid/particle ratios.^[^ ^27^ ^]^ The resultant capillary forces lead to the formation of liquid bridges and the creation of particle networks. As a result, the bulk rheological behavior of the suspension alters significantly from predominantly viscous or weakly elastic to highly elastic or gel‐like. This phenomenon can be observed in various particle/liquid systems, and despite being used frequently for functional ink formulation,^[^ 28, 29 ^]^ only one report is available on extrusion printing of graphene capillary suspension ink.^[^ ^30^ ^]^ Extrusion printable inks are amongst the most challenging types of inks and usually require considerable amounts of additives (>25 wt%). It has been shown that the addition of only 2 vol% octanol, as the immiscible solvent, to a 16.67 wt% aqueous graphene suspension is sufficient to form a gel that exhibits shear‐thinning behavior with high enough yield strength for extrusion printing.

Sinter‐free nanoparticle‐based inks (including 2D materials), even when formulated without additives, form films with relatively low compaction level,^[^ ^31^ ^]^ which results in poor electrical properties. To improve the interparticle charge transfer and overall conductivity of the network, mechanical compression or rolling techniques can be used. It has been shown that the conductivity of a graphene laminate can be increased by more than 50 times after one pass of compression rolling (conductivity of 4.3×10^4 ^S m^−1^ and sheet resistance of 3.8 Ω sq^−1^ (with a thickness of 6 μm)).^[^ ^7^ ^]^ In additive‐containing inks, mechanical compression would be even more beneficial since decomposition of the additives generates gases that can further increase the porosity of the films, especially in postprocessing methods with very high heating rates such as photonic annealing (Figure 1d).^[^ 19, 26 ^]^

**Figure 3:**
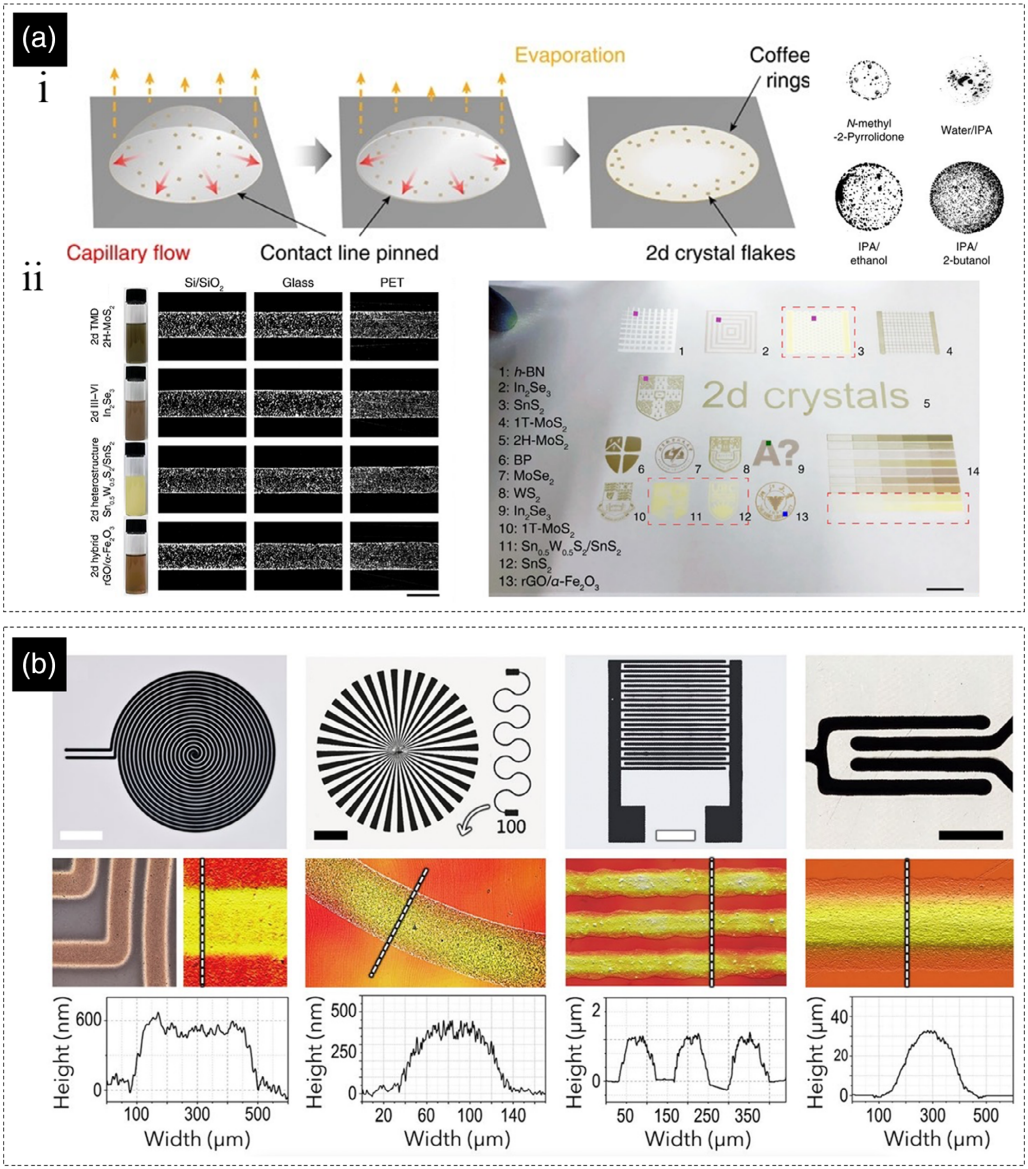
a) i) On the left, the coffee‐ring effect is schematically depicted and, on the right, the digital image of a dried droplet of several inks with different carrier solvent are shown; ii. Using a proper cosolvent system, coffee‐ring effect has been avoided and different 2D materials inks have been printed and various substrates (scale bar: 500 μm). a) Reproduced under the terms of the CC‐BY Creative Commons Attribution 4.0 International license (https://creativecommons.org/licenses/by/4.0).^[^ ^32^ ^]^ Copyright 2020, The Authors, published by American Association for the Advancement of Science. b) In high viscosity gel type inks printed with different printing methods and various substrates, coffee‐ring effect is not observed, and the profiles of the printed structures are usually semielliptical. Scale bars are 3, 3, 1.5, and 2 mm from left to right, respectively. Reproduced under the terms of the CC‐BY Creative Commons Attribution 4.0 International license (https://creativecommons.org/licenses/by/4.0).^[^ ^6^ ^]^ Copyright 2020, The Authors, published by Wiley‐VCH.

Drying and film formation behavior of the inks, especially in industrial scale printing/coating, are very important considerations for ink formulation. The shape of the profile of the printed tracks and structures can have significant impact on the performance of the printed devices. A rectangular profile is considered as an ideal case, but its realization in practice is very difficult and often a semielliptical profile would be acceptable for most of the applications. However, due to a very common phenomenon in drying particle‐based inks, namely the coffee‐ring effect, even realizing the semielliptical profile can often be very challenging. As shown in **Figure** **3**a, during the drying of a well‐wetting ink (with pinned edges), because of the higher evaporation rate of the solvent from the sides (due to higher surface area to volume ratio), an outward flow from the center of the printed structure toward its edges is generated to replenish the evaporated solvents. Such a flow can carry and accumulate the particles on the edge of the droplet and forms a ring‐like structure. This problem can be addressed by the application of multicomponent carrier solvents (cosolvent systems) for ink formulation. It has been recently shown that a binary mixture of IPA‐t‐butanol (90–10 vol%) can be used for uniform deposition of different 2D crystals and their derivatives.^[^ ^32^ ^]^ The disparity of evaporation rate of different components in the inks, surface tension, and compositional gradients from center to edges and gives rise to inward Marangoni flows, creating a more uniform redistribution of the particles (Figure 3a). The coffee‐ring effect is mostly observed in low‐viscosity inks as movement of particles in high viscosity solvents takes much longer time (and eventually film dries). The coffee‐ring effect can even be fully avoided in gel type inks, since the solvent is dispersed within the network of the particles and hence cannot carry them to the edges (Figure 3b).

One of the necessities in the electronic device industry is finding an appropriate process to develop and fabricate transistors as a backbone of an electric circuit. There is a broad range of research to design and fabricate printed different transistors instead of hybrid versions. For the fabrication of electrolyte‐gated transistors (EGTs), there are some electrical and chemical compatibility, sintering, and flexible substrate issues. Therefore, as compared with metal inks, such as silver‐based inks, which might react with strong oxidizing chemicals, graphene is the most suitable printable material, with an acceptable range of conductivity and chemical stability, and it can also go under process at lower temperatures. For fully IJP of electrolyte‐gated transistors, for the gate part, graphene flakes stabilized by EC in cyclohexanone and terpineol are used as ink. The composite solid polymer electrolyte is prepared by adding poly(vinyl alcohol) to dimethyl sulfoxide solvent. Then the mixture of these two solutions is used as the ink.^[^ ^33^ ^]^

A very recent attempt to fabricate a touch screen sensor based on a coating of liquid‐exfoliated graphene ink showed that the main and most crucial properties of transparent conductive electrodes are electrical performance, visual performance, and mechanical flexibility. These requirements were met by spray coating graphene onto the flexible substrate with an optical transmittance of 78%, a sheet resistance of 290 Ω sq^−1^, and no significant change in sheet resistance when bent up to a radius of 28 mm. Furthermore, the low level of attenuation and high signal‐to‐noise ratio (14 dB) allow for a multitouch operating mode. Also, in the same study, different solvents and methods were tested for graphene exfoliation, which has significant differences in the results. The obvious differences are related to the environmental issue (selection of solvent), yield, and size distribution of nanosheets.^[^ ^34^ ^]^

Another sensing application can be related to implementing label‐free and high‐sensitive graphene inks to get a response for quantitatively detecting biological species like SARS‐CoV‐2. For this, graphite flakes were exfoliated with a high‐shear mixer and stabilized with EC. Various steps of washing, redispersing, and filtration were applied for dispersion of graphene in terpineol to formulated aerosol‐jet‐printable ink. The fabricated sensors were heat treated at 350 °C for 30 min in a furnace to eliminate residual solvent. Aerosol‐jet‐printed (AJP) graphene‐based dipstick functionalized electrodes were incubated with a SARS‐CoV‐2 spike then the electrode surface was blocked with buffer until further testing.^[^ ^35^ ^]^ In **Table** **1**, some of the most promising graphene ink formulations for different printing and coating methods are summarized.

**Table 1:** Printing and coating graphene using common methods

Printing/Coating method	Ink composition	Post processing	Substrate	Remarks
Spray coating^[^ ^36^ ^]^	Graphene conc.: 10 g L^−1^	Spray coating at 120 °C	Al foil	Graphene was first exfoliated in NMP using wet jet milling and then transferred to EtOH‐H_2_O
	Carrier solvent: 50:50 vol% EtOH‐H_2_O			Sheet resistance: ≈40 Ω sq^−1^
	Stabilizing agent/Binder: Sodium carboxymethyl cellulose (0.5 g L^−1^)			Application: Electrochemical double‐layer capacitor
Spray coating^[^ ^37^ ^]^	Graphene conc.: 60 mg L^−1^	Spray coating at 120 °C and Annealing at 40 °C, 1 h	FTO‐coated glass	*ν*(ink) = 1.02 mPa s
	Carrier solvent: DMF			Semi‐transparent film (transmittance 44%)
	Stabilizing agent/Binder: ‐			Sheet resistance: Application: Dye‐sensitized solar cell
Spray coating^[^ ^25^ ^]^	Graphene/NC conc.: ≈5 g L^−1^	Various temp	Glass slides and polyimide	Spraying pressure
	Carrier solvent: 80:20 acetone/ethyl lactate			≈200 kPa. Substrate temp. 100 °C during spraying
	Stabilizing agent/Binder: ‐			Conductivity: ≈10 000–40 000 S m^−1^
				Application: Printed electronics
Blade coating^[^ ^23^ ^]^	Graphene conc.: 100 g L^−1^	Annealing at 30 °C, 40 min	Glass	Graphene nanoplatelets produced by high‐pressure microfluidization method (4% of the flakes <4 nm, and 4< rest (96%) <70 nm)
	Carrier solvent: Deionized water			Conductivity: ≈20 000 S m^−1^
	Stabilizing agent: Sodium deoxycholate (9 g L^−1^)			Application: Printed electronics
	Rheology modifier: Carboxymethylcellulose sodium salt (10 g L^−1^)			
Blade coating^[^ ^25^ ^]^	Graphene/NC conc.: 10% w/v	Various temperatures	Glass slides and polyimide	Graphene/NC is dispersed in ethyl lactate with excess acetone (≈2:1 acetone/ethyl lactate v/v) then removed by heating
	Carrier solvent: Ethyl lactate‐stabilizing agent/Binder: ‐			Conductivity: ≈10 000–40 000 S m^−1^
				Application: Printed Electronics
Slot‐die coating^[^ ^6^ ^]^	Graphene conc.: 0.5 vol%	No post processing	PET	Fully room temperature processed
	Carrier solvent: Propylene glycol			Sheet resistance: 3 Ω sq^−1^
	Stabilizing agent/Binder: ‐			Application: Transistor
Inkjet printing^[^ ^38^ ^]^	Graphene conc.: 0.11 g L^−1^	17 °C, 5 min	(HMDS)‐ Si/SiO_2_, and Si/SiO_2_	For 20:80 EG:NMP *Z *≈ 11.2
	Carrier solvent: 20:80 Ethylene glycol: NMP			For 80:20 EG:NMP *Z *≈ 2.8
	or 80:20 Ethylene glycol: NMP			Particles larger than 1 μm were filtered out to avoid clogging of the nozzle
	Stabilizing agent/Binder: ‐			Sheet resistance: ≈30 kΩ sq^−1^
				Application: Thin‐film transistors
Inkjet printing^[^ ^39^ ^]^	Graphene conc.: 0.62 g L^−1^	No postprocessing	PET	Graphene was exfoliated in 50:50 EtOH: H_2_O by sonication and concentrated with ultrahigh centrifugation
	Carrier solvent: 50:50 EtOH: H_2_O			Sheet resistance: 13 kΩ sq^−1^
	Stabilizing agent/Binder: ‐			Application: Printed electronics
Inkjet printing^[^ ^3^ ^]^	Graphene conc.: 1.6 g L^−1^	No postprocessing	Coated PET	Graphene was exfoliated in NMP by sonication and concentrated with ultrahigh centrifugation (6.22 g L^−1^), for NMP‐based in *Z* ≈ 17
	Carrier solvent: NMP			Conductivity: G/MoS_2_ ≈3000 S m^−1^
	Stabilizing agent/Binder: ‐			Application: Printed photodetectors
Inkjet printing^[^ ^40^ ^]^	Graphene conc.: 0.1 g L^−1^ GW, 0.36 g L^−1^ GEth	≈80 °C for 2 h	Si/SiO2	Graphene was exfoliated in deionized water with sodium deoxycholate
	Carrier solvent: Water–isopropyl alcohol and water–ethanol			for NMP‐based in *Z* ≈ 12
	Stabilizing agent/Binder: ‐			Resistance: ≈11 kΩ
				Application: Terahertz saturable absorbers
Inkjet printing^[^ ^41^ ^]^	Graphene conc.: 2 g L^−1^	No postprocessing	Plastic and paper	Graphene was exfoliated in deionized water with PS1 by sonication
	Carrier solvent: Water + propylene glycol (10:1 by mass)			For modified ink *Z* ≈ 20
	Stabilizing agent/Binder: Pyrenesulfonic acid sodium salt (PS1) (0.5 g L^−1^)/Xanthan gum (0.1 g L^−1^)			Sheet resistance: 10–1 kΩ sq^−1^
	Surface tension modifier agents: Triton x‐100 ≥ 0.06 g L^−1^			Application: Photosensors, logic memory devices
Inkjet printing^[^ ^42^ ^]^	Graphene conc.: 0.40 g L^−1^	No post processing	Si/SiO_2_	Graphene was exfoliated in PVP‐IPA solution
	Carrier solvent: IPA			For PVP‐IPA‐G ink *Z* ≈ 9.48
	Stabilizing agent/Binder: Polyvinyl pyrrolidone (0.15 g L^−1^ in IPA)			Sheet resistance: ‐
				Application: humidity sensor
5Inkjet printing^[^ ^43^ ^]^	Graphene conc.: 0.42 g L^−1^	400 °C for 30 min	FTO/glass	Particles larger than 1μ m were filtered out to avoid clogging of the nozzle
	Carrier solvent: IPA			For graphene‐PVP in IPA ink *Z* ≈ 9.6
	Stabilizing agent/Binder: polyvinyl pyrrolidone (0.15 g L^−1^ in IPA)			Sheet resistance: 600 Ω sq^−1^
				Application: Dye‐sensitized solar cells
Inkjet printing^[^ ^21^ ^]^	Graphene conc.: 2.4 wt% solids (≈3.4 g L^−1^)	250–350 °C anneal for 30 min	(HMDS)‐ Si/SiO_2_, and Si/SiO_2_	Graphene was exfoliated in ethanol/ethyl cellulose by probe ultrasonication and concentrated with salt addition method
	Carrier solvent: Cyclohexanone/terpineol 85:15 Stabilizing agent/Binder: Polyvinyl pyrrolidone (0.15 g L^−1^ in IPA)			Resistivity: 4 mΩ cm
				Application: Flexible electronics
Inkjet printing^[^ ^25^ ^]^	Graphene conc.: ≈10 g L^−1^	Various temperature	Glass slides and polyimide	Graphene/NC is dispersed in ethyl lactate with excess acetone (≈2:1 acetone/ethyl lactate v/v) then removed by heating
	Carrier solvent: 75:15:10 ethyl lactate/octyl acetate/ethylene glycol diacetate			Conductivity: ≈10 000–40 000 S m^−1^
	Stabilizing agent/Binder:‐			Application: Printed electronics
Inkjet printing^[^ ^44^ ^]^	Graphene conc.: ≈0.40–0.93 g L^−1^	400 °C for 30 min	Polyimide and PET	Fully room temperature processed
	Carrier solvent: Isopropyl alcohol			Sheet resistance: 300 Ω sq^−1^
	Stabilizing agent/Binder: polyvinylpyrrolidone 0.15–1.2 g L^−1^			Application: thermoelectric devices
Inkjet printing^[^ ^45^ ^]^	Graphene conc.: 1% w/v graphene/EC (4% w/v solid)	Preannealing at 80 °C for 60–180 min then annealed with intense pulsed light	PET and PEN	Inks were filtered with a 3.1 μm glass fiber syringe to prevent nozzel clogging
	Carrier solvent: 85:15 v/v cyclohexanone/terpineol			Conductivity: 25 000 S m^−1^
	Stabilizing agent/Binder: Polyvinylpyrrolidone 0.15–1.2 g L^−1^			Application: Flexible electronics
Aerosoljet printing^[^ ^46^ ^]^	Graphene + Binder conc.: 3.1 g L^−1^	450 °C	Si/SiO_2_ wafers with 300 nm SiO_2_	Graphene was exfoliated in ethanol/ethyl cellulose by a high‐shear mixer and concentrated with salt addition method
	Carrier solvent: Cyclohexanone/Terpineol 92.5:7.5			Sheet resistance: 1.64 kΩ sq^−1^
	Stabilizing agent: Ethyl cellulose			Application: Miniaturized printed electronic
Aerosoljet printing^[^ 47, 48 ^]^	Graphene + Binder conc.: 30 g L^−1^	35 °C in air	Polyimide	Graphene was exfoliated in acetone/nitrocellulose by a high‐shear mixer and concentrated with salt addition method
	Carrier solvent: 1:9 v/v mixture of dibutyl phthalate and ethyl lactate			Conductivity: 1–7 × 10^4^ S m^−1^
	Stabilizing agent/Binder: Nitrocellulose			Application: Sensors
Flexographic printing^[^ ^49^ ^]^	Graphene conc.: 25 g.L^−1^	Unclear	Polyimide	Graphene was exfoliated in ethanol/ethyl cellulose by a high‐shear mixer and concentrated with salt addition method
	Carrier solvent: 4:1 v/v ethanol/terpineol			Sheet resistance: <1 kΩ sq^−1^
	Stabilizing agent: Ethyl cellulose (3.6% w/v)			Application: Sensors
Gravure printing^[^ ^50^ ^]^	Graphene + Binder conc.: 10% w/v	25 °C, 30 min	Polyimide	Graphene was exfoliated in ethanol/ethyl cellulose by a high‐shear mixer and concentrated with salt addition method
	Carrier solvent: Terpineol			Conductivity: −10 000 S m^−1^
	Stabilizing agent/Binder: Ethyl cellulose			Application: Flexible electronics
Screen printing^[^ ^51^ ^]^	Graphene conc.: ≈53.3 g L^−1^	10 °C, 5 min	PET and Paper	Expanded graphite was exfoliated in isopropanol and 60:40 copolymer of *N*‐vinyl‐2‐pyrrolidone and vinyl acetate using a high‐shear mixer
	Carrier solvent: Di(propylene glycol) methyl ether			Sheet resistance: 30 Ω sq^−1^
	Stabilizing agent/Binder: 60:40 copolymer of *N*‐vinyl‐2‐pyrrolidone and vinyl acetate (3:1 graphene wt.)			Application: Printed flexible electronics
Screen printing^[^ ^52^ ^]^	Graphene conc.: 8% w/v	30 °C, 30 min	Polyimide	Graphene was exfoliated in ethanol/ethyl cellulose by a high‐shear mixer and concentrated with the salt addition method
	Carrier solvent: Terpineol			Conductivity: 1.86 × 10^4^ S m^−1^
	Stabilizing agent/Binder: Ethyl cellulose (12% w/v)			Application: Flexible printed electronics
Extrusion printing^[^ ^26^ ^]^	Graphene conc.: 2.8% w/v	Substrate at 6 °C during the printing and intense flash lamp annealing for removing the binder at room temperature	Glass and plastics	Graphene was exfoliated in acetone/nitrocellulose by a high‐shear mixer and concentrated with the salt addition method
	Carrier solvent: Ethyl lactate			Sheet resistance: 0.1–5 kΩ sq^−1^
	Stabilizing agent/Binder: Nitrocellulose (5.2% w/v)			Application: Microsupercapacitors
Extrusion printing^[^ ^30^ ^]^	Graphene conc.: 16.62 wt%	35 °C, 30 min	Free‐standing object	Commercial graphene nanoplatelets with avg. thickness of 8 nm and lateral size of 5 μm were used
	Carrier solvent: Water (+2 vol% octanol)			Conductivity: 2370 S m^−1^
	Stabilizing agent/Binder: Sodium carboxymethyl			Application: 3D printing
	cellulose (1:4.2 graphene wt.)			

## 3 Semiconductive Inks

### 3.1 Black Phosphorus (BP)

BP has gained a lot of attention since 2014 after being exfoliated in a liquid phase and used as an efficient field‐effect transistor (FET).^[^ 53, 54, 55 ^]^ Phosphorus (P) atoms are bound to three neighboring atoms with sp^3^ hybridized orbitals but with a puckered honeycomb structure since atoms are not on the same plane. Held together by weak vdW forces, the interlayer distance between BP monolayers is 0.53 nm in bulk form.^[^ 53, 55, 56 ^]^ Depending on the number of layers, BP can also have a tunable direct bandgap (ranging between 0.3 and 2.0 eV), which is placed between graphene (zero bandgap) and TMDs (1.0–2.0 eV).^[^ ^57^ ^]^ This unique property leads to much higher carrier mobility (≈1000 cm^2^ V^−1^ s^−1^) in comparison to TMDs.^[^ ^53^ ^]^ Overall, besides the abovementioned properties, characteristics like high surface area, good biocompatibility, and low cytotoxicity make BP a very attractive material in a wide variety of fields such as biosensors,^[^ ^58^ ^]^ energy storage devices,^[^ ^59^ ^]^ optoelectronics, FET,^[^ ^60^ ^]^ and catalysis.^[^ ^61^ ^]^

BP can be synthesized in various forms, including bulk crystals, nanosheets, and quantum dots. Delamination of high‐quality BP nanosheets has been reported using almost all common techniques for the exfoliation of 2D materials such as micromechanical,^[^ 62, 63 ^]^ electrochemical exfoliation,^[^ ^64^ ^]^ and LPE methods.^[^ 65, 66 ^]^ **Table** **2** summarizes various LPE methods for producing BP monolayers. Most studies have recommended using organic solvents to impede the BP degradation induced by O_2_ and H_2_O. However, due to the environmental and health‐related considerations, several attempts have been made to obtain stable dispersions of BP using aqueous solutions, and some of them have also been very successful.^[^ 67, 68 ^]^ Despite its exciting properties, BP is not chemically stable in the ambient atmosphere.^[^ 69, 70 ^]^ The exposure of BP to O_2_ and H_2_O leads to the formation of P_*x*_O_*y*_ and phosphoric acid is formed, respectively. Therefore, lots of studies have been carried out to improve the chemical instability of PB by controlling its physicochemical properties, mainly using doping techniques.^[^ ^71^ ^]^ Comprehensive discussions on doping BP using different methods and dopants have been published in other works.^[^ 72, 73, 74, 75 ^]^

**Table 2:** Summary of dispersion media and exfoliation condition for BP

Dispersion Media	LPE Conditions	Remarks	References
ACN	Method: Bath sonication (360 W)	Acetonitrile (ACN) solvothermal treatment at 200 °C for 24 h	[76]
	Initial concentration: 1 mg mL^−1^	Synthesis in inert atm	
	Sonication duration: 30–240 min	Lateral dimensions: >10 μm	
	Centrifugation: 1500 rpm for 20 min	Thickness of layers: 2 nm	
	Centrifugation: 4000 rpm for 45 min		
Water	Pretreatment: Grounding	Flexible lithium‐ion battery application	[77]
	Method: Probe sonication (950 W)		
	Initial concentration: 1–10 mg mL^−1^	Max yield: 0.4 mg mL^−1^	
	Sonication duration: 30–300 min	Thickness of layers: >5.2 nm (10 layers)	
	Centrifugation: 1500–5000 rpm for 30 min		
Nine different Ionic liquids (ILs)	Pretreatment: Grounding	Yield: 0.95 mg mL^−1^	[78]
	Method: Bath sonication (100 W)	Thickness: 3.58 to 8.90 (few layers)	
	Initial concentration: 3 mg mL^−1^		
	Sonication duration: 24 h		
NMP	Method: Bath sonication (40 kHz)	Humidity sensor application	[79]
	Initial concentration: 1 mg mL^−1^ Sonication duration: 8 h		
	Centrifugation: Cascade centrifugation	Different thickness and layers based on centrifugation steps	
Water + IPA (different ratios)	Method: Bath sonication (120 W)	Exfoliation under Ar atm	[80]
		Average flake size: 118–165 nm	
	Initial concentration: 0.4 mg mL^−1^		
	Sonication duration: 6 h		
	Centrifugation: 4000 rpm for 15 min	Thickness: the majority are monolayers or bilayers	
DMF	Method: Probe sonication	BP dispersion with a concentration of 6.5 mg mL^−1^ (PS/NMP)	[81]
	Initial concentration: 20 mg mL^−1^	Thickness of layers: 12 nm (60 sheets)	
	Sonication duration: 4 h	Later dimension: Average 250 nm	
	Centrifugation: 2500 rpm for 60 min, 5000 rpm for 90 min		
DMF and DMSO	Method: Sonication (130 W)	Thickness for DMF less than 5 nm	[82]
	Initial concentration: ≈0.02 mg mL^−1^	Thickness for DMSO less than 15–20 nm	
	Sonication duration: 15 h		
	Centrifugation: 2000 rpm for 30 min		
Water	Method: Sonication	Yield: ≈0.02 mg mL^−1^	[83]
	Initial concentration: 0.5 mg mL^−1^	Thickness: 2 nm (4 layers)	
	Sonication duration: 8 h		
	Centrifugation: 1500 rpm for 10 min		
anhydrous acetone	Method: Sonication (300 W)	Supercapacitor applications	[84]
	Initial concentration: 1 mg mL^−1^	Drop casting	
	Sonication duration: 10 h		
	Centrifugation: 2000 rpm for 60 min		
Water Additive: SDS	Method: Probe sonication (70 W)	FET fabrication	[85]
	Initial concentration: 1 mg mL^−1^	Effective exfoliation: Ultrasonication in deoxygenated water stabilized with surfactants	
	Sonication duration: 8 h	Thickness of layers: 4.5 nm	
	Centrifugation: 7500 rpm for 2 h, 14 000 rpm for 2 h		
CHPa)	Method: Probe sonication (750 W)	Gas sensing application	[86]
	Initial concentration: 2 mg mL^−1^	Lateral dimension: ≈1 μm	
	Sonication duration: 5 h	Yield: 1 mg mL^−1^	
	Centrifugation: Cascade centrifugation		
NMP	Method: Bath sonication (820 W)	Spin coating at 6000 rpm	[87]
	Initial concentration: 5 mg mL^−1^	Lateral dimension: 200 nm	
	Sonication duration: 24 h	Thickness of layers: 3−3.5 nm	
	Centrifugation: 1500 rpm for 45 min		
NMP	Method: Probe sonication (30 W)	FET devices	[88]
	Initial concentration: 1 mg mL^−1^	Drop casting and electron beam lithography	
	Sonication duration: 1 h	Yield: 0.4 mg mL^−1^	
	Centrifugation: 500–15 000 rpm for 10 min	Thickness of layers: 16–128 nm	
Different solvents	Pretreatment: Grounding	Lateral dimension: 50 nm −50 μm	[66]
Preferred: benzonitrile	Method: Bath sonication	Yield: 0.11 mg mL^−1^	
	Initial concentration: 0.5 mg mL^−1^	For scale‐up using combination of sonication and shear mixer	
	Sonication duration: (8–10) × 99 min		
	Centrifugation: Various		
NMP	Method: Shear mixer	Thickness of layers <4.5 nm (<10 layers)	[65]
	Initial concentration: 1 mg mL^−1^		
	Sonication duration: 30 min		
	Centrifugation: Cascade centrifugation		

a) *N*‐cyclohexyl‐2‐pyrrolidone.

BP can be successfully exfoliated in numerous solvents, including *N*‐cyclohexyl‐2‐pyrrolidone (CHP), NMP, acetonitrile (ACN), isopropyl alcohol (IPA), and 2‐methoxy ethanol (2‐ME). However, it has been found that the exfoliation yield is considerably higher for the high‐boiling‐point solvents.^[^ ^89^ ^]^ Simulation results have shown that NMP molecules prefer to intercalate between BP layers, facilitating the delamination of bulk BP.^[^ ^90^ ^]^ In addition to forming stable suspensions, CHP can provide a solvation shell around BP and improve its oxidation resistance, therefore CHP is an appealing solvent for ink formation. Although by adjusting the concentration of the CHP‐based ink, its viscosity (10 cP) can also be perfectly tuned to meet the requirements of the IJP,^[^ ^91^ ^]^ neither CHP nor NMP are good choices for IJP, as their high boiling point can cause numerous problems including severe coffee‐ring effect.

Therefore, considering the lower exfoliation yield of the low boiling point solvents, exfoliation in NMP (or CHP) and transferring the exfoliated nanosheets to a low boiling point solvent seem to be a practical solution. Following this approach, Jun et al.^[^ ^89^ ^]^ have first separated the exfoliated nanosheets from NMP by filtration and then after drying have formulated an inkjet printable ink by redispersing the dried powder in 2‐ME (using sonication). To avoid the coffee‐ring effect, which is a common problem in single solvent inks, they have optimized the concentration of the ink. Printing with inks with the concentration of 1 mg mL^−1^ has led to uniform distribution of BP nanosheets across the printed patterns avoiding a noticeable coffee ring formation.

Formulating inks using binary cosolvent systems is proven to be an efficient strategy for addressing the coffee‐ring effect. When a secondary solvent is added to an ink, upon its drying, the concentration of the higher‐boiling‐point solvent will be higher on the edges and lower in the inner sections of the film. This may result in a temperature gradient in the droplet due to the latent heat of vaporization and hence a recirculating surface tension gradient, inducing a recirculating Marangoni flow that can help to redistribute the particles more evenly across the film. In this regard, Hasan and his group have formulated a BP inkjet printable ink by first transferring the exfoliated sheets (from NMP) to IPA and then adding 2‐butanol (10 vol%) to suppress the coffee‐ring effect. This solvent system has several other advantages over NMP‐based inks, especially for the formulation of inkjet printable inks. The NMP‐based inks, due to their relatively higher surface tension (e.g., compared to IPA) and low viscosity, usually have a high Ohnesorge number (*Z* > 14), which is not suitable for IJP. However, using the IPA‐2 butanol system, inks with *Z* ≈ 10 (*D* = 22 μm) have been formulated, which are well within the optimal *Z* value range for stable jetting.^[^ ^92^ ^]^

Although there is an instability issue of BP under ambient conditions, it still can be used for device fabrication. Various modifications in the solution process steps make it possible to exfoliate BP nanosheets, and based on the selected printing method, inks can be reliably transferred and incorporated in optoelectronic and/or photonic devices. Suppressing the coffee ring problem and using binder‐free ink without any substrate pretreatment can increase the consistency and uniformity of the process and final products. As mentioned, BP is an ideal material for visible and near‐infrared optoelectronics, including photodetectors. Using an inkjet‐printed BP layer, such a photodetector, not only shows >10 times enhancement in detection performance at 450 nm but also extends the detection range up to 1550 nm. Besides promising results, encapsulation with parylene‐C makes the printed BP more stable against long‐term (>30 days) oxidation.^[^ ^92^ ^]^

### 3.2 Transition Metal Dichalcogenides (TMDs)

One of the most widely studied groups of 2D materials with naturally accruing layered crystals is the TMDs family. These materials are characterized by the general formula of MX_2_, where M^4+^ is a metal cation (such as Mo, W, V, Nb, Ti, and Zr), and X^2−^ is a chalcogenide anion. The monolayer of TMDs consists of positively charged layers of metal cations (M) sandwiched between two negatively charged layers of chalcogenide (X) anions.^[^ 93, 94 ^]^ Although the *M*–X intralayer bonds are very strong (ionic covalent), the MX_2_ nanosheets are stacked and held together by weak vdW attractions. As a result, their parent layered crystal can be exfoliated into a single and or few layers using similar methods that are used for the exfoliation of graphene.^[^ 95, 96, 97, 98, 99 ^]^

**Figure 4:**
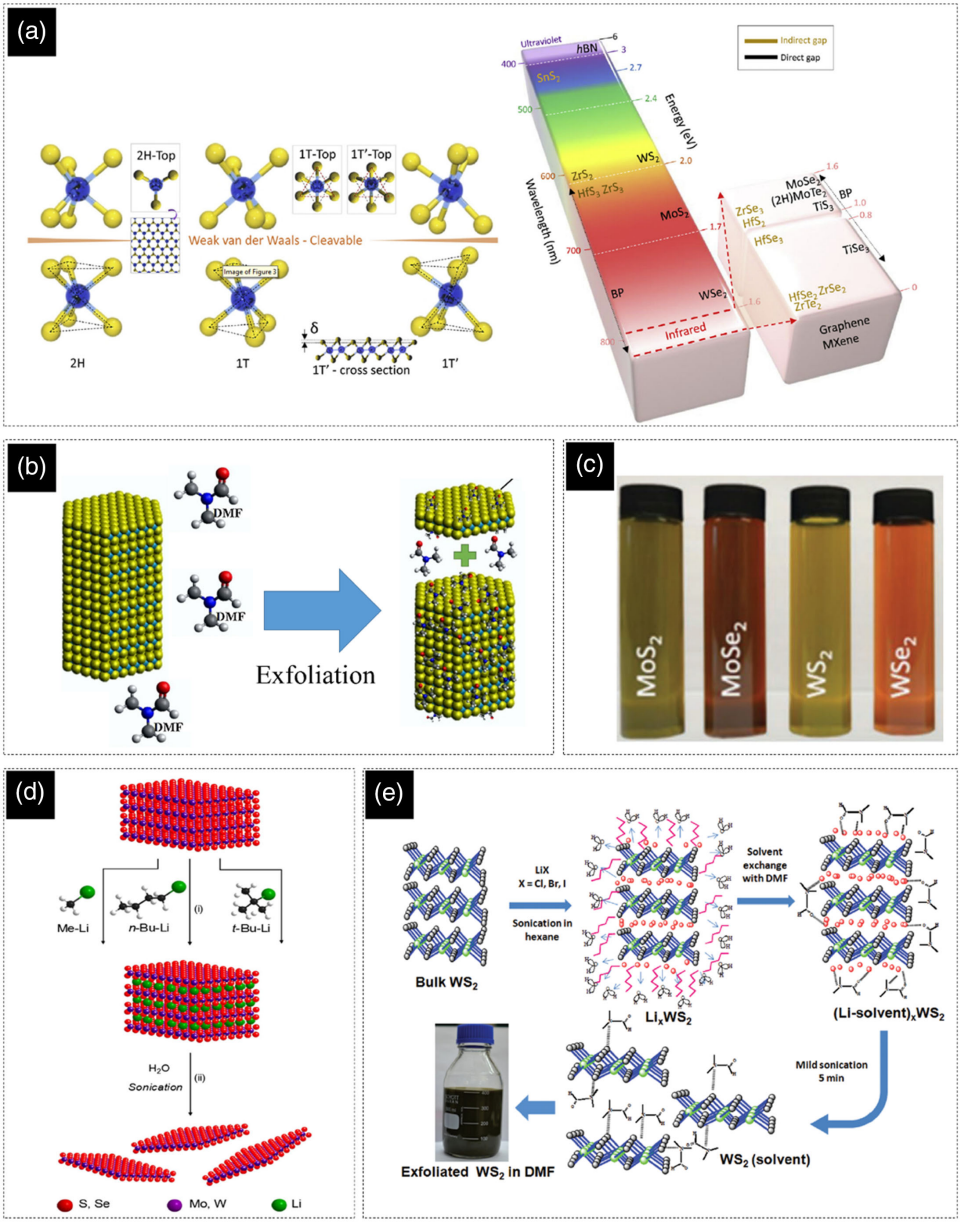
a) Typical structures and bandgap of transition metal dichalcogenides. The color in the column represents the wavelength of the bandgap. Reproduced with permission.^[^ ^105^ ^]^ Copyright 2016, Elsevier Ltd. b) Schematic of direct exfoliation procedure for WS_2_ with DMF. Reproduced with permission.^[^ ^112^ ^]^ Copyright 2019, Elsevier Ltd. c) Exfoliated and dispersion of some important TMD nanosheets in water. Reproduced with permission.^[^ ^113^ ^]^ Copyright 2021, Wiley‐VCH. d) Sonication‐assisted exfoliation using organic lithium‐based solutions such as methyllithium (Me‐Li), n‐butyllithium (n‐Bu‐Li), and tert‐butyllithium (t‐Bu‐Li). Reproduced with permission.^[^ ^114^ ^]^ Copyright 2015, Elsevier Ltd. e) Schematic representation of lithium halide‐assisted synthesis of few‐layered WS_2_, the process is starting with sonication in hexane solvent followed by exchanging hexane to DMF. Reproduced with permission.^[^ ^65^ ^]^ Copyright 2016, Royal Society of Chemistry.

In contrast to the single layer of graphene, the TMD monolayer shows novel physical and chemical properties that strongly differ from their bulk forms. The transformation from bulk to monolayer structure causes a transition from indirect‐to‐direct bandgap due to thickness‐induced quantum confinement (**Figure** **4**a).^[^ 100, 101, 102, 103, 104, 105 ^]^ Moreover, the nanosheets of TMDs show polymorphism related to different symmetries of the coordination of the metal ion. The most common phases are 1T, 2H, and 3R based on tetragonal, hexagonal, and rhombohedral symmetries.^[^ ^106^ ^]^ Decreasing the thickness and number of the layers provides not only a high surface area and abundant surface‐active sites but also bandgap modulation possibility over a wide range, enabling novel applications in numerous fields such as catalysis,^[^ ^107^ ^]^ optoelectronics,^[^ ^108^ ^]^ sensing,^[^ ^109^ ^]^ energy storage,^[^ ^110^ ^]^ and biomedicine.^[^ ^111^ ^]^

As mentioned earlier, thanks to their structural similarities, most of the techniques for the exfoliation/synthesis of graphene can also be used for TMDs, for example, methods such as mechanical exfoliation,^[^ ^115^ ^]^ LPE,^[^ ^116^ ^]^ chemical vapor deposition,^[^ ^117^ ^]^ magnetron sputtering,^[^ ^118^ ^]^ and hydrothermal synthesis.^[^ ^119^ ^]^ However, due to their significantly higher exfoliation energy (surface energy),^[^ ^98^ ^]^ delamination of the TMDs is more difficult than graphene, which is a big challenge for the production of high concentration suspensions and ink formulation. Since the exfoliation method (e.g., the dispersion medium) can drastically affect the chemical/structural properties of the TMDs (will be discussed later),^[^ ^120^ ^]^ and hence their performance, the exfoliation/synthesis of the 2D TMDs requires further attention. Therefore, before discussing the printing/coating of this group of 2D materials, their LPE, which is usually the starting point for ink formulation, will be briefly reviewed.

LPE of TMDs can be mainly categorized into direct exfoliation (Figure 4b,c) and lithium‐intercalation‐assisted exfoliation (Figure 4d,e).^[^ 105, 112, 113, 114, 121, 122 ^]^ For direct exfoliation, the bulk material is dispersed in a suitable solvent, usually nitrogen‐containing solvents such as NMP, which enable long‐time stable and relatively high‐concentration suspensions.^[^ ^123^ ^]^ Direct exfoliation in solvents does not involve the formation of an intermediate intercalated compound. The solvent molecules may enter interlayer galleries of the layered solids, but their interactions are not strong enough to form a stable intercalated phase.^[^ 124, 125 ^]^

The shear force necessary for LPE can be applied with different methods. A comparison between magnetic stirring, shear mixing, and probe sonication showed that probe sonication produces the most optimal dispersions, among others.^[^ ^126^ ^]^ So far, most of the studies have been mainly focused on developing dispersion media for an optimum exfoliation process, while the sonication process itself has received much less attention. Generally, simple ultrasonic cleaning baths are used for treating a noticeable amount of material, since these devices do not adequately control transmission power and temperature, nanosheet disintegration, or aggregation cannot be avoided.^[^ ^127^ ^]^ However, new studies have emerged in recent years that emphasize the effects of ultrasound physics and sonication parameters on the efficacy of the exfoliation of layered dichalcogenides and the sonochemical transformations of the used solvents. Sonication parameters may critically affect the thickness, sizes, and other properties of the products (e.g., chemical structure) formed from bulk TMDs or other layered materials.^[^ ^128^ ^]^

High local temperatures and pressures accompanying the cavitation phenomenon have been reported as the driving force for nanosheet exfoliation. When cavitation bubbles implode close to the surface of the particles, high‐speed jets of liquids or shock waves may develop and create a physicochemical effect on the surface.^[^ 129, 130 ^]^ Indeed, two main consequences of MX_2_ sonication are: 1) exfoliation, i.e., the separation of the vdW bonded layers; and 2) fragmentation, hence chopping of the bigger particles into smaller ones by breaking covalent bonds.^[^ 131, 132 ^]^ Parameters such as sonotrode shape, depth of the sonotrode, which is immersed in a liquid, ultrasound intensity, pressure and temperature of the reaction, material concentration, and solvent density have substantial effects on the process and the final results.^[^ ^127^ ^]^

As mentioned before, the choice of the solvent in LPE plays a crucial role in the exfoliation process. The quality and the quantity of the exfoliated flakes can be controlled by the physicochemical properties of the used solvents, such as solubility, surface energy, and boiling point. The roles of the solvent(s) during the exfoliation are as follows: they transfer the sonotrode acoustic power, decrease the mixing energy between nanosheets and the liquid, and stabilize the delaminated sheets by giving a steric wall to prevent reaggregation.^[^ ^125^ ^]^

Nonetheless, due to the high surface energy of the TMDs, the chance of reaggregation is higher in pure solvents, which necessitates the use of stabilizing agents.^[^ ^98^ ^]^ Sodium cholate is one of such stabilizing agents that has shown great promise^[^ ^98^ ^]^ for the production of highly concentrated dispersions of various 2D materials such as MoS_2_, WS_2_, MoSe_2_, MoTe_2_, NbSe_2_, and NiTe_2_.

Green and eco‐friendly solvents are particularly desirable for printing and large‐area coating methods, but exfoliation of TMDs in such solvents (e.g., water or ethanol) is difficult and yields low concentration suspensions. Using cosolvent systems (e.g., water/ethanol mixtures) is a promising alternative for stabilizing agent‐based strategies and offers the possibility to formulate additive‐free inks.^[^ ^133^ ^]^ It is worth mentioning that when choosing the dispersion system for the LPE, it should be considered that chemical reactions can occur upon exfoliation of the TMDs in certain solvents. For instance, it has been shown that the exposure of MoS_2_ to methanol (e.g., in a water/methanol system) can lead to the formation of sulfur vacancies.^[^ ^120^ ^]^

The Li‐intercalation‐exfoliation is the other main type of exfoliation method for the production of TMDs, in which their parent crystals are immersed in a solution containing lithium ions such as hexyllithium or n‐butyllithium under inert and dry conditions for a few days. During this process, Li intercalates into the interlayer spaces of TMDs and reacts to form compositions such as Li_*x*_MX_2_. Then, this compound is sonicated in a water bath where Li reacts with water, leading to the evolution of hydrogen gas. These reactions lead to the delamination of TMDs into individual layers.^[^ ^106^ ^]^ It should be noted that during the intercalation‐assisted exfoliation of TMDs, their bulk 2H structure is usually distorted, and a phase transition from semiconductive 2H to metallic 1T may occur.

Since the ion intercalation methods are time consuming and involve harmful chemicals such as lithium compounds, electrochemical exfoliation methods have been recently adopted to overcome these limitations. By applying and controlling an external potential, radicals/ions are generated in the electrolyte and aggregate between the layers of TMDs. Ion accumulation and the generation of gases then cause the expansion/detachment of TMD layers.^[^ ^134^ ^]^ One big challenge in electrochemical exfoliation of the TMDs is the low conductivity of their bulk material, as most of the applied potential will be used to overcome the huge resistance. To address this problem, conductive additives are usually added to the powders of the TMDs’ parent crystal, and conductive monoliths are made and used for their electrochemical exfoliation.^[^ ^135^ ^]^

#### 3.2.1 Tungsten Disulfide (WS_2_)

Tungsten disulfide (WS_2_) is one of the most extensively studied types of semiconducting TMDs, which has been used in numerous applications such as FETs, phototransistors, photovoltaics, and gas sensors.^[^ 136, 137, 138 ^]^ The LPE of WS_2_ has been so far reported in many different dispersion media (**Table** **3**), but because of cost and environmental considerations, aqueous suspensions and inks are highly preferable to their organic counterparts. Nevertheless, water‐based ink formulation is very challenging, mainly due to its high surface tension and low viscosity. Therefore, in the majority of the previous reports on printing and coating of WS_2_, the ink formulation has been based on either solvent exchange, use of additives, or a combination of both.

**Table 3:** Summary of dispersion media and exfoliation condition for WS_**2**_

Dispersion Media	LPE Conditions	Remarks	References
DMSO and Water (1:1)	Pretreatment: Oxygen plasma (15 min) + SF6 plasma treatment kept dispersion in DMSO and Water for 72 h	Lateral size 45 μm	[139]
(Additive: SnO_2_ QDs (0.1 mL of 0.05 m solution))	Method: Probe sonication (400 W)		
	Initial concentration: 0.04 mg mL^−1^		
	Sonication duration: ‐ hour		
	Centrifugation: 600 g for 90 min		
NMP	Method: Bath sonication (40 kHz)	Sensing application	[140]
	Initial concentration: 1.5 mg mL^−1^		
	Sonication duration: 3 h		
	Centrifugation: 3000 rpm for 15 min		
Ethanol:Water	Method: Bath sonication	NO_2_ gas sensing	[133]
	Initial concentration: 24 mg mL^−1^		
	Sonication duration: 6 h		
	Centrifugation: 4000 rpm		
IPA:Water (3:7)	Pretreatment: Soaking in water (5 min)	EHD printing of photodetector	[141]
	Method: Bath sonication (37 kHz)	Solvent exchange and ink (propylene glycol:water (8:92))	
	Initial concentration: ‐ mg mL^−1^	Additives: Triton X‐100 and xanthan gum	
	Sonication duration: 6 h	Yield: 1 mg mL^−1^	
	Centrifugation: Cascade centrifugation		
Triacetin, Triethyl Citrate, Ethanol:water (45:55)	Method: Bath sonication	Electrochemical sensing application	[142]
	Initial concentration: 7.5 mg mL^−1^	Drop‐casting technique Yield: 0.0798, 0.0646, and 0.004 mg mL^−1^ in triacetin, triethyl citrate, and ethanol/water, respectively	
	Sonication duration: 2 h		
	Centrifugation: 4000 rpm for 45 min		
C:T/EC solution mixture	Method: Probe sonication	Capacitive structure application	[143]
	Initial concentration: 30 mg mL^−1^	Inkjet printing	
	Sonication duration: 6 h	Ink: Cyclohexanone:terpineol (7:3) solution	
	Centrifugation: ‐	Additives ethylcellulose (2.5 wt%)	
		C/T/EC has a viscosity of ≈12 cP	
DMSO and Water (1:1)	Pretreatment: Plasma in different gases and different powers (same duration)	Photodetector application	[144]
Oxygen‐free Water	Method: Probe sonication (400 W)	Drop‐casting technique	
	Initial concentration: 10 mg mL^−1^		
	Sonication duration: 4 h		
	Centrifugation: 2000 rpm for 90 min		
DMSO:Water (1:1 Molar ratio)	Pretreatment: (NH_4_)_2_CO_3_ water solution + microwave	High‐quality nanosheets average lateral size of 400 nm and thickness of layers of 2.33 nm.	[145]
	Method: Bath sonication (400 kHz)	Washing and transferring to ethanol	
	Initial concentration: 2 mg mL^−1^		
	Sonication duration: 2 h		
	Centrifugation: ‐		
Water + DMSO	Pretreatment: 1) Keep dispersion of WS_2_ in DMSO and water mixture for 72 h; and 2) Oxygen plasma for 3 min	Photodetectors application	[146]
	Method: Probe sonication (400 W)	Drop‐casting technique	
	Initial concentration: 10 mg mL^−1^	Water/DMSO average lateral 350–400 nm thickness of layers 2 nm	
	Sonication duration: 4 h	Water/DMSO (O_2_‐plasma) average lateral size 500–600 nm thickness of layers 2 nm	
	Centrifugation: 2000 rpm for 90 min		
Aqueous solution of sodium cholate (2 g L^−1^)	Pretreatment: 1 h probe sonication	Transistor application	[147]
	Method: Probe sonication(400 W)	Airbrush spraying	
	Initial concentration: 30 mg mL^−1^	Mean flake length ≈415 nm and mean layer number ≈20–30	
	Sonication duration: 7 h		
	Centrifugation: Cascade centrifugation		
DMF (Additive: 5 mL NaPF6 dissolved in 1 m DEGDME)	Method: Probe sonication (400 W)	Washing and transferring WS_2_ to isopropyl alcohol (IPA) or DMF	[148]
	Initial concentration: 1 mg mL^−1^	Gas sensing application	
	Sonication duration: 9 h	Inkjet printing	
	Centrifugation: 8000 rpm for 10 min	Flake size distribution 50–400 nm and mean flake size ≈165	
Ethanol:DI water (1:1)	Method: Probe sonication (750 W)	Solar cells application	[149]
	Initial concentration: 6 mg mL^−1^	Yield: 0.6 mg mL^−1^	
	Sonication duration: 6 h	Spin coating at 1500 rpm for 60 s	
	Centrifugation: 6000 rpm		
Aqueous solution of Epigallocatechin gallate (EGCG)a)	Method: Probe sonication (950 W)	Lithium‐sulfur batteries application	[150]
	Initial concentration: 2 mg mL^−1^	Approximately five monolayers with a uniform particle size distribution of 100–200 nm	
	Sonication duration: 12 h	Washing and transferring to water	
	Centrifugation: 1500 rpm for 30 min, then supernatant 10 000 rpm for 30 min		
Ethanol:Water (7:3)	Method: Sonication (150 W)	Photocatalytic application	[151]
	Initial concentration: 8 mg mL^−1^	Yield: 0.2 mg mL^−1^	
	Sonication duration: 8 h	Thickness of the flake <4 nm (4–6 layers)	
	Centrifugation: 3344 rpm for 30 min	Lateral dimensions range: 300–700 nm	
NMP	Method: Bath sonication	Inkjet printing	[152]
	Initial concentration: 8 mg mL^−1^	0.75 mL of the WS_2_/NMP dispersion + 0.75 mL of mono ethylene glycol (MEG) to raise the viscosity	
	Sonication duration: 10 h		
	Centrifugation: 1200 g for 60 min		
Binary systems	Method: Bath sonication (379 W)	Conceteration of dispersion in IPA‐water > EtOH–water > ACN–water > MeOH–water	[153]
	Initial concentration: 1 mg mL^−1^	Highest yield: Isopropanol:water (20:80)	
low‐boiling‐point solvents with Water (IPA, Ethanol, Methanol, CAN)	Sonication duration: 10 h		
	Centrifugation: 3000 rpm for 20 min		
C:T/EC solution	Method: Probe sonication	Ethylcellulose as a viscosity modifier (12 cP)	[154]
	Initial concentration: 30 mg mL^−1^	Spin coating (1) 800 rpm for 60 s; and 2) 2000 rpm for 20 s)	
	Sonication duration: 3 and 6 h		
	Centrifugation: ‐	Inkjet printing	
Combinations of isopropanol, ethylcellulose, and terpineol	Method: Probe sonication	Drop‐casting technique	[155]
	Initial concentration: 5 mg mL^−1^		
	Sonication duration: 99 and 297 min		
	Centrifugation: 2000 rpm for 30 min		
N‐vinylpyrrolidone (NVP)	Method: Probe sonication (400 W)	Washing and transferring to ethanol	[100]
	Initial concentration: 3 mg mL^−1^	Redispersing in isopropanol (IPA)	
	Sonication duration: 4 h	Thickness of the layers: 0.7–2 nm (1–3 layers)	
	Centrifugation: 5000 and 10 000 rpm for 10, 15, and 20 min	Lateral dimensions ≈ 100 nm	
		Spin coating	

a) (35 mL; EGCG, 0.01–2 mg mL^−1^).

**Figure 5:**
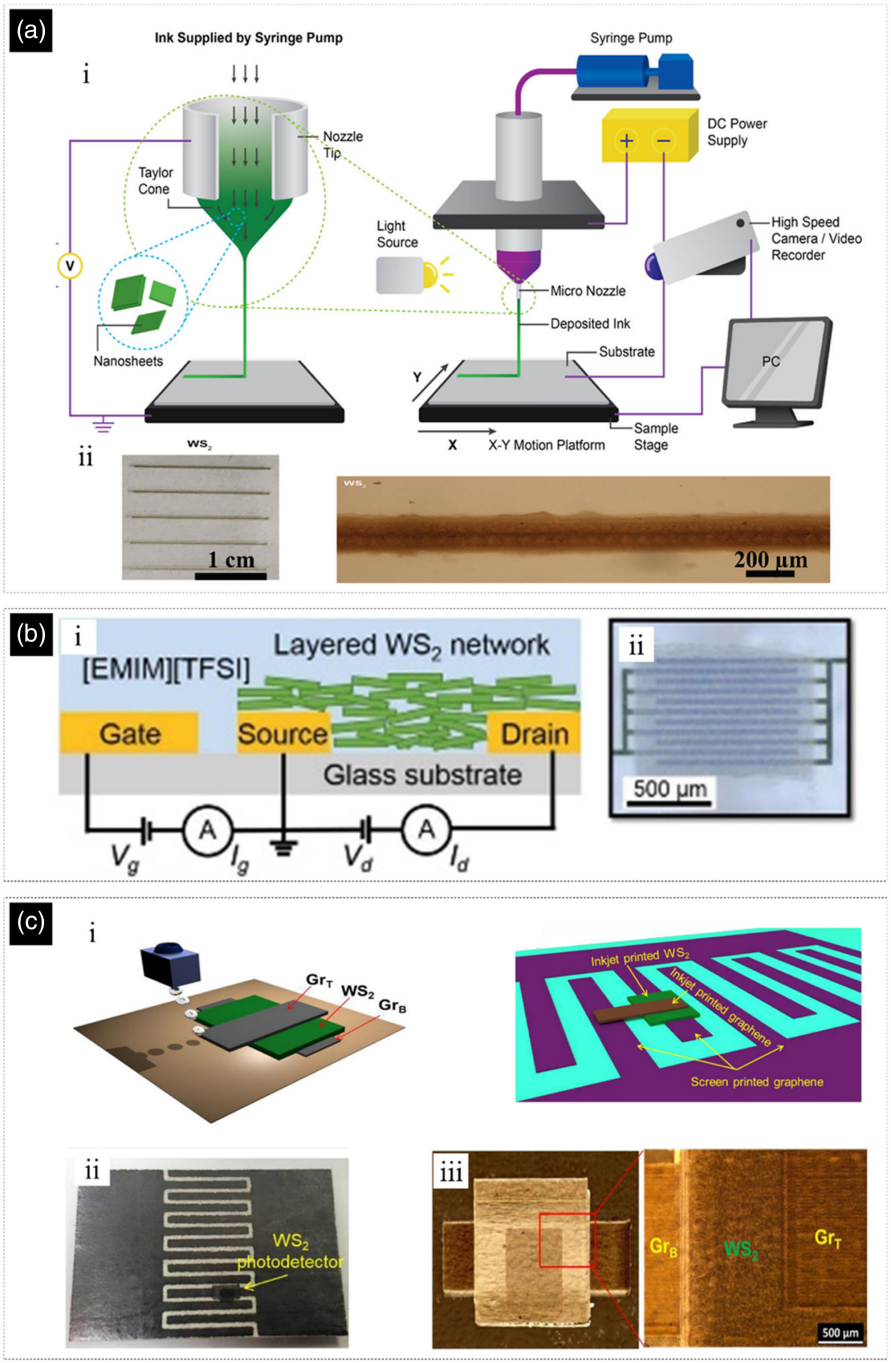
a) i) Schematic illustration of an electrohydrodynamic (EHD) printing setup. A high voltage is applied between the nozzle and the substrate, forcing the liquid out of the nozzle to form a Taylor cone. ii) Graphene, MoS_2_, and WS_2_ printed lines on a glass substrate. iii) Magnified image of the printed lines. a) Reproduced with permission.^[^ ^156^ ^]^ Copyright 2020, American Chemical Society. b) i) Schematic of the layered WS_2_ network transistor structure. ii) Optical image of deposited WS_2_ nanosheet network on interdigitated electrodes. b) Reproduced with permission.^[^ ^147^ ^]^ Copyright 2019, The Authors, published by Wiley‐VCH. c) i) Schematic of different printed layers of a photodetector, the graphene top (GrT), and bottom (GrB) electrodes and the WS_2_ active layer. ii) Image of the inkjet‐printed WS_2_ photodetector integrated with screen‐printed graphene line. iii) Image of the device printed onto PEL P60 paper. c) Reproduced under the terms of the CC‐BY Creative Commons Attribution 3.0 Unported license (https://creativecommons.org/licenses/by/3.0).^[^ ^157^ ^]^ Copyright 2019, IOP Publishing Ltd.

As noted, using cosolvent systems is one of the main strategies for exfoliating 2D materials. For instance, the water/IPA mixture is a good choice for the exfoliation of WS_2_ due to its suitable surface tension and other physicochemical properties. It has also been shown that soaking the bulk powder of WS_2_ in water/IPA (7:3) prior to the exfoliation process can activate the powder and improve the exfoliation yield. However, a water/IPA‐based ink would suffer from bad printability and various drying‐related issues such as the coffee ring effect. These issues can be mostly addressed by transferring the exfoliated nanosheets to a propylene glycol/water (8:92) mixture. The solvent exchange step also provides an opportunity to properly adjust the concentration of the ink for a more efficient printing/coating. To further improve the printability of the ink for the electrohydrodynamic printing of a fully printed photodetector (**Figure** **5**a), a small amount of xanthan gum as a binder and Triton X‐100 as a surface tension modifier have also been added to the ink.^[^ ^156^ ^]^

Pyrene sulfonic acid (PS) derivatives are very efficient stabilizing agents for the dispersion of numerous 2D materials. Triton X‐100, which is a nonionic surfactant, is a good choice for lowering the surface tension of the inks that are stabilized by PS derivatives, as it does not disrupt their electrostatic stabilization.^[^ ^41^ ^]^ To minimize the adverse effects of the additives on the electronic properties of the functional materials, usually different concentrations of stabilizing agents are used for the exfoliation and the ink formulation. For example, in a recent report on the production of an aqueous WS_2_ inkjet printable ink,^[^ ^41^ ^]^ first WS_2_ (1.5 g) was exfoliated in 500 mL water containing 0.5 g of 1‐pyrenesulfonic acid sodium (PS1) salt and then the excess PS1 has been separated by ultracentrifugation. The sediments are then redispersed in water containing ≥0.06 mg mL^−1^ Triton X‐100 and ≥0.1 mg mL^−1^ xanthan gum. A small amount of propylene glycol has also been added (10:1, water:propylene glycol) to increase the viscosity and bring the Ohnesorge number's (Z) closer to the acceptable range (1 < *Z* < 14).^[^ ^157^ ^]^ Similarly, in another work for spray coating WS_2_ for the fabrication of an electrolyte‐gated field effect transistor (Figure 5b), WS_2_ has been first exfoliated in sodium cholate but then sedimented and redispersed in DI water. To remove the residues even more thoroughly, the coated films have also been soaked in DI water for 12 h. In spite of all these efforts, WS_2_ that is a *p*‐type semiconductor has exhibited n‐type behavior, which has been attributed to residues of sodium cholate.^[^ ^147^ ^]^

The organic WS_2_‐based inks are very similar in composition to those of graphene. A mixture of cyclohexanone and terpineol (7:3) has been frequently used as a carrier solvent for WS_2_ ink formulation for lots of printing and coating methods. The addition of EC (2.5 wt%) can help with both stabilizing the nanosheets and adjusting the rheological properties for an inkjet printable ink formulation (viscosity: ≈12 cP). The effectiveness of this approach and composition for stable ink formulation has also been demonstrated for various WS_2_‐based composite inks (with graphene and other nanomaterials).^[^ 126, 143 ^]^ Like most other 2D materials, NMP can effectively exfoliate WS_2,_ and although the obtained suspension can be directly used for printing, adding a small amount of mono ethylene glycol (MEG) can increase the viscosity from 1.69 to 2.5 cP, and significantly improve its printability for IJP.^[^ ^152^ ^]^

Another WS_2_ nanosheet printing example is IJP of a WS_2_ layer on a screen‐printed graphene layer for fabricating a battery‐free wireless photosensor (Figure 5c). WS_2_ nanosheets were obtained from sonication of an aqueous dispersion, whereas exfoliated graphene flakes in NMP were produced first by a high shear mixing at 8000 rpm for 2 h followed by an ultrasonication step for 24 h. The flakes were then re‐dispersed in ethylene glycol for ink preparation. Developing this combination of materials and methods enabled functional RF electronic device fabrication.^[^ ^157^ ^]^

Among the recent advances, gas sensing applications have been receiving more attention. MOSFET‐type sensor with an inkjet‐printed layer of WS_2_ demonstrates a special sensitivity (increasing or decreasing the drain current) when the sensor is exposed to specific gases. The sensor shows high selectivity toward NO_2_ gas among four target gases (NO_2_, H_2_S, NH_3_, and CO_2_).^[^ ^148^ ^]^

#### 3.2.2 Molybdenum Disulfide (MoS_2_)

Bulk MoS_2_ is an indirect bandgap semiconductor (1.2 eV) that changes to a direct bandgap (1.9 eV) upon exfoliation to single layers. The thickness and number of the layers significantly affect the size of the bandgap since the quantum confinement effect can be observed in such size ranges.^[^ 158, 159 ^]^ Therefore, screening the particle size after the exfoliation process and before the ink formulation is of great importance. In an interesting investigation with surprising results, it has been found that the selection of the starting bulk material for LPE of MoS_2_, which was chosen from six sources, including high‐quality large crystals and fine powder, has either no or minor effect on the quality and quantity (yield) of the final product.^[^ ^101^ ^]^

**Figure 6:**
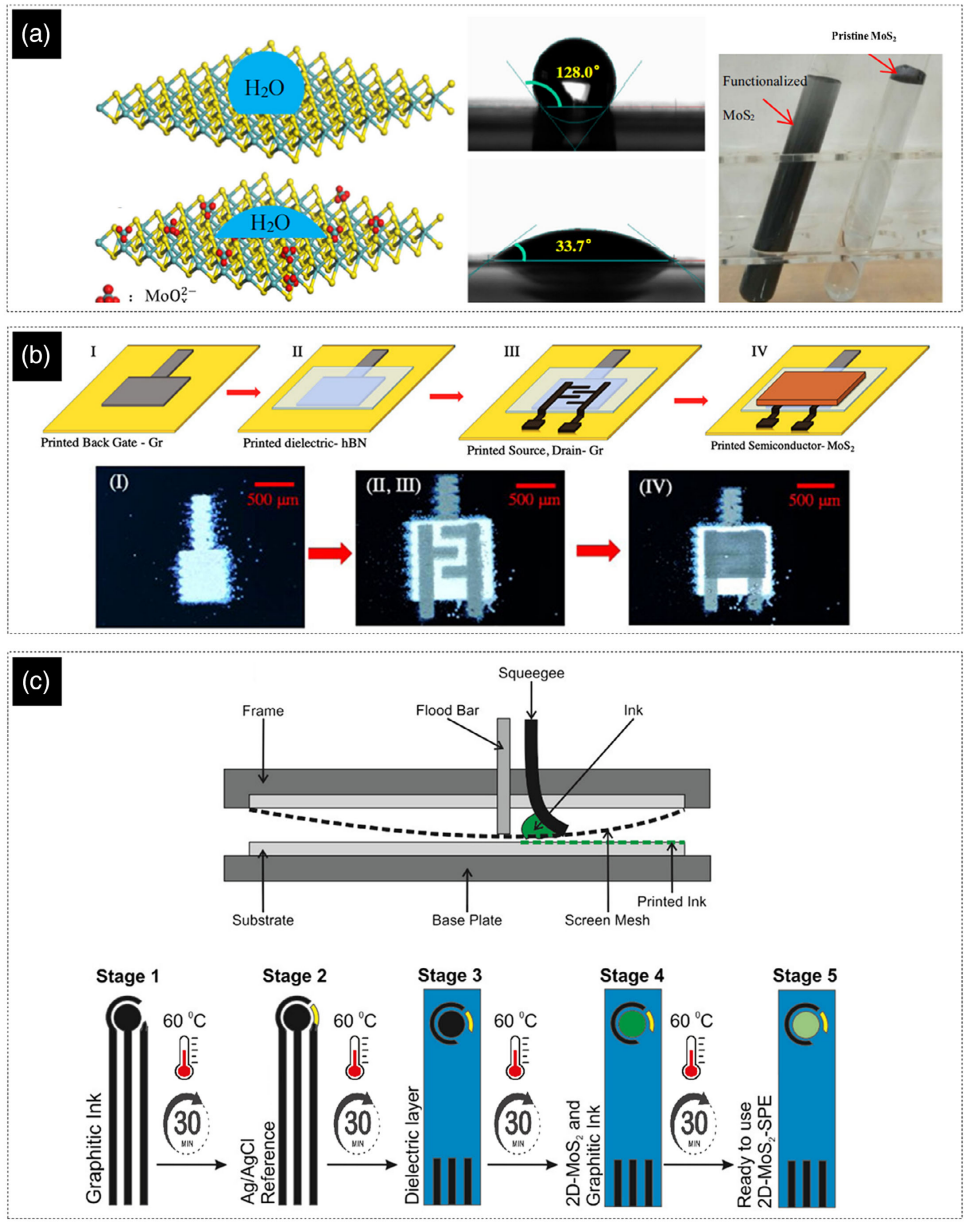
a) Functionalization of TMDs affects the wettability of nanosheets. Different contact angles and dispersion behavior are obvious in pure water without any surfactants for pristine and treated MoS_2_ nanosheets. Reproduced with permission.^[^ ^162^ ^]^ Copyright 2019, Elsevier Ltd. b) Schematic and inkjet‐printed FET fabrication with graphene—bottom gate layer, h‐BN layer on top of graphene layer as source and drain and MoS_2_ layer on top. Reproduced with permission.^[^ ^163^ ^]^ Copyright 2020, The Authors, published under license by AVS. c) Screen‐printing process and the necessary steps for fabricating a high throughput of 2D‐MoS_2_‐SPEs. Reproduced with permission.^[^ ^164^ ^]^ Copyright 2017, American Chemical Society.

Ion intercalation is one of the major techniques for the exfoliation of MoS_2_, especially for its large‐scale production, which can be done in water and other solvents such as ethanol, methanol, and isopropyl alcohol.^[^ ^160^ ^]^ However, as it has been mentioned earlier, ion intercalation can cause phase transformation from 2H‐MoS_2_ (thermodynamically stable indirect bandgap semiconductor) to 1T‐MoS_2_ (octahedral metallic phase). This process is also associated with the generation of energetic defect sites in the nanosheets and, consequently, a hydrophobic to hydrophilic transition in the surface properties of the MoS_2_ (**Figure** **6**a). It is worth mentioning that the 2H to 1T transition provides a unique opportunity for the functionalization of the 1T‐MoS_2_ nanosheets with fluoroelastomers that can be used for the fabrication of stretchable devices such as solid‐state supercapacitors.^[^ 161, 162 ^]^

Since Li‐ion intercalation is a slow and time‐consuming process, special strategies may be implemented to accelerate it significantly using different energy sources such as ultrasonication,^[^ ^165^ ^]^ microwave irradiation,^[^ ^166^ ^]^ or solvothermal conditions.^[^ ^167^ ^]^ In the case of ultrasonication, implosion of cavitation bubbles causes an increase in the local temperature and pressure that leads to the degradation of butyllithium hexamers into monomers (or any other lithium‐containing compounds) and enhancing the electron transfer to MoS_2_ and facilitating the insertion of the Li from two aspects. One is forcing the Li to intercalate into the interlayer spaces, and the second is based on the fragmentation and chopping of the sheets and increasing the probability of insertion.^[^ 127, 168 ^]^ Obtaining flakes with large lateral dimensions of up to 100 μm is possible by using different intercalating agents such as the double salt of potassium sodium tartrate KNaC_4_H_4_O_6_·4H_2_O; however, the produced nanosheets will have a partial oxidization and a distorted 1T structure.^[^ ^169^ ^]^

In addition to the transformation of the indirect to direct bandgap, several other properties of MoS_2_ are also changed upon its delamination to a single or few layers. For instance, the magnetic characteristics of the nanoscale MoS_2_ sheets would also alter, exhibiting strong ferromagnetism due to the presence of edge spins on the edges of the nanosheets.^[^ 170, 171 ^]^ Selection and controlling of the lateral dimensions of the flakes, in addition to the deposition technique, heavily depend on their ultimate application. Small nanosheets perform better in catalytic applications than big nanosheets, which are targeted for electronics and composites. This is due to the fact that the catalytically active sites are usually located at the edges of the nanosheets, and smaller nanosheets have a higher density of these sites and edge atoms.^[^ ^127^ ^]^

As mentioned earlier, the low concentration of the suspension of 2D materials is a big obstacle to the formulation of commercial inks and efficient printing. One of the main strategies for addressing this problem is dispersing the 2D material in a dispersion medium (**Table** **4**) that gives the highest yield and then transferring the exfoliated particles to another solvent (or solvent system) that has a smaller quantity and also offers better printability. In this regard, an inkjet printable ink has been formulated by first exfoliating the nanosheets in DMF (with the aid of EC) and transferring them to terpineol (20 times less solvent) by evaporation of the DMF.^[^ ^172^ ^]^ The viscosity and the surface tension of the ink have been further adjusted for IJP by the addition of a small amount of ethanol.

**Table 4:** Summary of dispersion media and exfoliation condition for MoS_2_

Dispersion Media	LPE Conditions	Remarks	References
Aqueous solution of the P123a) Surfactant in Water	Method: Probe sonication (≈100 W)	Visible‐light photocatalysis application	[173]
	Initial concentration: 10 mg mL^−1^	Lateral dimensions 50–90 nm	
	Sonication duration: 17 h	Thickness of layers 1.5–2 nm (single and few layer)	
	Centrifugation: 1500 rpm for 5 min		
Aqueous solution	Method: Probe sonication (500 W)	Calculation of concentration, lateral size, and layer number based on the optical spectra	[101]
*C*_sodium cholate_ = 2 g L	Initial concentration: 7.5 mg mL^−1^		
	Sonication duration: 5 h		
	Centrifugation: Cascade centrifugation		
Isopropanol (IPA) + Saltsa) Solution	Method: Bath sonication (200 W)	Washed with water and finally transferred to IPA	[174]
	Initial concentration: ‐ mg mL^−1^	Yield: 0.240 mg mL^−1^	
	Sonication duration: 2 h		
	Centrifugation: 3000 rpm for 20 min		
NMP solution (NaOH (0.25 mg mL^−1^))	Method: Bath sonication (200 W)	Sodium ion battery application	[175]
	Initial concentration: 1 to 50 mg mL^−1^	Concentration 0.6 mg mL^−1^ (for 1 mg mL^−1^)	
	Sonication duration: 2 h	Average nanosheets size 1 and 9 nm, (1–9 layers)	
	Centrifugation: 2000 rpm for 30 min		
Pure Solvents such as IPA, Ethanol, Methanol, Water	Pretreatment: Solvothermal functionalization	Hydrophobic and uncharged surface transformation to negatively charged and hydrophilic surface	[162]
	Method: Bath sonication	<10 layers and 15% monolayer	
	Initial concentration: 10 mg mL^−1^	Yield: water 0.17 mg mL^−1^, methanol 0.23 mg mL^−1^, ethanol 0.46 mg mL^−1^, and IPA 1.01 mg mL^−1^	
	Sonication duration: 12 h		
	Centrifugation: 4000 rpm for 15 min		
Ethanol	Method: Probe sonication	Thickness of 30 nm,13 nm, and 0.75 nm for the sonication time of 2,15, and 45 h, respectively.	[176]
Pyrene‐assisted LPE	Initial concentration: 0.2 mg mL^−1^		
	Sonication duration: Diverse periods		
	Centrifugation: 1500 rpm for 5 min		
Keratin solution	Method: Bath sonication (250 W)	Thickness of nanosheets 3–4 nm	[177]
	Initial concentration: ‐ mg mL^−1^	Size of the nanosheets is roughly 100 nm	
	Sonication duration: 48 h	Optimum weight concentration ratio between MoS_2_ and the WKb) is ≈5:3.	
	Centrifugation: 5000 rpm for 45 min	Better exfoliation of WK at the low concentration level (1 mg mL^−1^)	
Polybutadiene + Toluene/NMP	Method: Bath sonication	Washing 3 times with toluene	[178]
	Initial concentration: ≈4 mg mL^−1^	Yield: 9 mg mL^−1^	
	Sonication duration: 6 days	Layer thickness: 3.3 nm (≈5 layers)	
	Centrifugation: 5000 then 14 000 rpm for 60 min	Lateral dimensions: 100–700 nm	

a) Potassium ferrocyanide, potassium sodium tartrate, sodium tartrate;

b) Wool keratin.

The transferring process can also be done from a high boiling point exfoliation solvent to a low boiling point carrier solvent (for ink formulation). In this case, nanosheets are separated from the exfoliation dispersion by ultrahigh‐speed centrifugation and then redispersed in the carrier solvent using a short/mild sonication process. Following this approach, an additive‐free aerosol jet printable ink (MoS_2_ concentration: 0.5 mg mL^−1^) has been produced by transferring the exfoliated MoS_2_ from NMP to a mixture of IPA and 2‐butanol (9:1 vol%).^[^ ^179^ ^]^

Like graphene and other TMDs, the cyclohexanone/terpineol (7:3) system proved to be a successful and frequently used composition for MoS_2_ ink formulation. Viscosity can be adjusted by the addition of EC (2.0 wt%), for example, for IJP. In this report, to ensure the continuity of the printed film and proper charge transfer through the network of the particles, overlayer printing of up to 20 passes has also been used.^[^ ^163^ ^]^

IJP provides an alternative route to a cost‐effective scaled‐up production of FETs to form high functionality devices with a wide variety of applications. With this method, all the different electric parts (conductor, semiconductor, and dielectric) of FETs are constructed with 2D‐layered materials. Controlling the flow of electrons or holes from source to drain is an important factor in any kind of transistor. This parameter depends on the size and shape of the channel region and the conductivity of the bottom gate. To accommodate these factors, graphene is selected as the gate, h‐BN, and MoS_2_ as a dielectric and semiconductor part, respectively. The printing sequences are shown in Figure 6b.^[^ ^163^ ^]^

Fabricating an electrode for the oxygen reduction reaction (ORR) electrodes is one of the most promising applications of the MoS_2_ nanoflakes obviating the need of high‐cost platinum. The process is based on the binding of electronegative oxygen atoms to the electropositive molybdenum atoms at the edge sites and shows electrocatalytic ORR properties. A screen printable commercial graphitic‐base ink was used with the modification of adding 2D‐MoS_2_ nanoflakes with different masses up to 40 wt%. The addition of MoS_2_ causes some changes in the viscosity of the formulated ink, which complicates the process of screen printing. As shown in Figure 6c, optimized conditions can alleviate this problem. The merit of screen‐printing is that reproducible films can be fabricated on a mass‐producible scale.^[^ ^164^ ^]^

#### 3.2.3 Tungsten Diselenide (WSe_2_)

Similar to most other semiconducting 2D materials, the exfoliation of bulk WSe_2_ is associated with a transition from an indirect (1.2 eV) to direct bandgap (1.7 eV) semiconductor.^[^ ^180^ ^]^ High intrinsic charge carrier mobility and the size and the position of the band edges make WSe_2_ a great choice for electronics (e.g., an active layer of transistors) and energy storage/conversion applications (e.g., hole transport layer in solar cells). LPE of the WSe_2_ in both aqueous (using stabilizing agents) and organic solvents have proven to be very successful (**Table** **5**). NMP is one of the main solvents, but low‐boiling point solvent mixtures such as IPA/water (30:70) have also shown great promise, especially when considering their lower costs and environmental hazards. The size of the solvent molecules and the surface energy of the dispersion media (optimum: 28 mN m^−1^) are two important considerations when choosing the dispersion system.^[^ ^180^ ^]^

**Table 5:** Summary of dispersion media and exfoliation condition for WSe_2_

Dispersion Media	LPE Conditions	Remarks	References
IPA + Water	Method: Probe sonication (60 W)	Photoelectric applications	[181]
	Initial concentration: 1 mg mL^−1^	Thickness of nanosheet 6–14 nm (8–20 layers)	
	Sonication duration: 1.5 h		
	Centrifugation: 3000 rpm for 10 min		
NMP	Pretreatment: Ground WSe_2_ in NMP for 2 h	NO_2_ sensors application	[182]
	Method: Probe sonication (400 W)	Washing with water and transferring to ethanol	
	Initial concentration: 2.5 mg mL^−1^	Drop cast of WSe_2_ dispersed in ethanol	
	Sonication duration: 20 h	Lateral dimensions: ≈200 nm	
	Centrifugation: 1500 rpm for 10 min, 8000 rpm for 20 min	Thickness of layers 4.2 nm (6 layers)	
IPA + Water	Method: Bath sonication (40 kHz)	Matching the ratio of surface tension components of co‐solvents	[183]
	Initial concentration: 3 mg mL^−1^		
	Sonication duration: 4 h		
	Centrifugation: 4000 rpm for 10 min		
NMP	Method: Bath sonication (35 kHz)	Concentrated via interface assisted extraction	[6]
	Initial concentration: 50 mg mL^−1^	Washing with ethanol	
	Sonication duration: 85 h	Different printing methods	
	Centrifugation: 3500 rcf for 30 min	Ink formulation: Solvent exchange	
Anhydrous ethanol	Method: Probe sonication (500 W)	NO_2_ sensors application	[184]
	Initial concentration: 4 mg mL^−1^	Drop casting	
	Sonication duration: 10 h	Thickness of layers: about 5 nm	
	Centrifugation: Cascade centrifugation		
NMP	Pretreatment: Ground WSe_2_ in NMP	Piezoresistive sensor application	[185]
	Method: Bath sonication (40 kHz)	Dip coating on paper, sintering at 200 °C to remove NMP	
	Initial concentration: 5 mg mL^−1^	Thickness: 4–5 layers	
	Sonication duration: 6 h		
	Centrifugation: 4000 rpm for 30 min		
Ethanol	Method: Bath sonication (300 W)	All‐optical switching application	[186]
	Initial concentration: 1 mg mL^−1^	Thickness: 4.1 nm (about 6 layers)	
	Sonication duration: 10 h		
	Centrifugation: 5000 rpm for 60 min, 12 000 rpm for 40 min		
Water	Method: Probe sonication (450 W)	Large‐area electronics application	[187]
	Initial concentration: 10 mg mL^−1^		
	Sonication duration: 10 h	Treatment with supercritical carbon dioxide (SC‐CO_2_) + sodium deoxycholate (NaDC) aqueous solution	
	Centrifugation: 3000 rpm for 15 min, 13 000 rpm for 15 min	Washing with water to remove residual NaDC	
		Ink formulation: Dispersing WSe_2_ nanosheets in hexylamine and transferring to the	
		hexane/ethylene glycol mixed solution	

So far, several techniques have been developed for improving the exfoliation of WSe_2,_ but one of the few works, which described a method that is very effective and yields highly uniform and phase‐pure 2H‐WSe_2_ semiconducting nanosheets, consists of pretreating the bulk WSe_2_ crystals with supercritical carbon dioxide (SC‐CO_2_). In this method, the as‐received WSe_2_ powder is stirred in a high‐pressure chamber (10 MPa) for a short time (30 min) in liquified CO_2_ at 55 °C. After depressurizing the system, the SC‐CO_2_‐treated WSe_2_ is exfoliated (by sonication) in water with the aid of sodium deoxycholate. While stabilizing agents such as sodium cholate or sodium deoxycholate can significantly improve the water dispersibility of WSe_2_^[^ ^98^ ^]^ and hence the ink formulation process, for most applications, the impurities should be removed thoroughly, making the whole printing process inefficient.^[^ ^180^ ^]^

As already stated, the products of the LPE methods usually have a very broad size distribution. Considering that the bandgap of 2D semiconductors can change significantly by slight changes in the number of layers in each flake (e.g., 0.5 eV for going from 1 to 6 layers), it is necessary to screen and separate the flakes for specific applications. For instance, such a big difference in the size of the bandgap of different flakes in the active layer of a transistor can act as charge carrier traps and drastically affect the device's performance. Few‐layered flakes (>6) are preferred to single layers in such applications (e.g., optoelectronic) since their bandgap is the same as the bandgap of the bulk form of the material. For instance, Kelly et al. have first exfoliated the WSe_2_ in NMP and then separated the particles comprising less than 6 layers with the cascade centrifugation method. Then by redispersing the size‐screened flakes in NMP (1.5 mg mL^−1^), an inkjet printable ink was obtained.^[^ ^4^ ^]^

Another challenge is the formulation of high viscosity inks for high‐throughput printing. Increasing the viscosity to such high levels requires large amounts of additives, which is very detrimental to semiconducting 2D materials. The same ink formulation strategy for the formulation of graphene‐based vdW inks can be used here as well. Using this technique, WSe_2_ nanosheets exfoliated in NMP are concentrated using an interface‐assisted extraction method and used for the formulation of gel‐type inks with suitable rheological properties for screen‐ and extrusion printing. Considering the morphological variations between the products of different exfoliation methods, it is suggested that the yield strength of the gel (which is a better representation of the rheological properties of the gel) is a better criterion for ink formulation and obtaining reproducible printing results than the ink concentration.^[^ ^6^ ^]^

**Figure 7:**
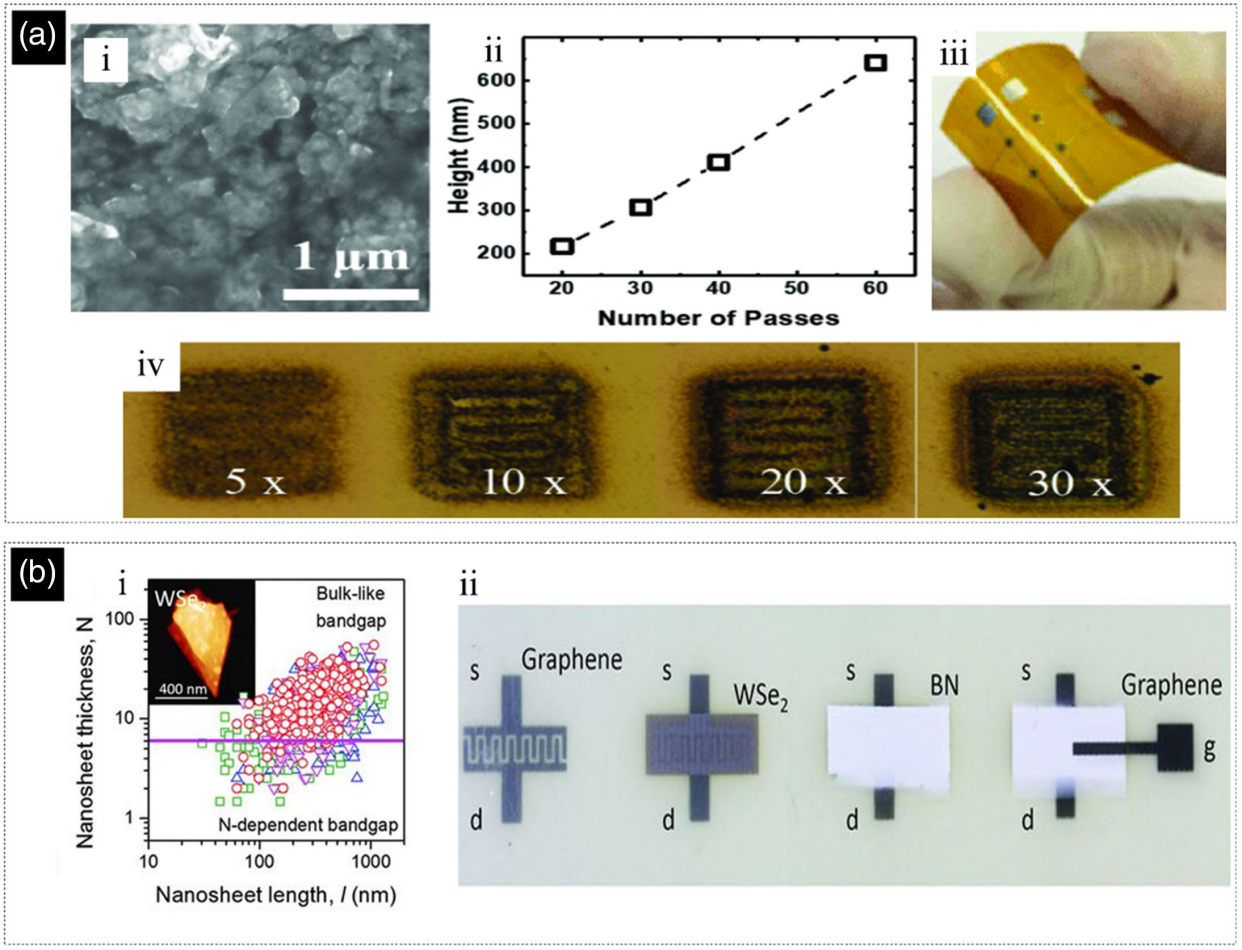
a) i) SEM image of the WSe_2_ surface layers, ii) diagram of thickness vs number of printing passes, iii) image of the flexible WSe_2_‐RRAM, and iv) optical microscopy images of the printed WSe_2_ layer with various number of printing passes. a) Reproduced with permission.^[^ ^188^ ^]^ Copyright 2019, IEEE. b) i) Plot of nanosheet length versus thickness (layer number *N*). The horizontal line approximately separates thinner nanosheets with a typical AFM image. ii) Photographs of the printing steps. From left to right: Graphene source (s) and drain d) electrode (*t* ≈ 400 nm); the WSe_2_ channel (*t* ≈ 1 mm, *L* = 200 mm, *w* = 16 mm); the BN separator (*t* ≈ 8 mm); and finally, the graphene gate (g, *t* ≈ 400 nm). b) Reproduced with permission.^[^ ^4^ ^]^ Copyright 2017, American Association for the Advancement of Science.

One of the applications of WSe_2_‐based inks is related to fully printed resistive random access memory (RRAM) on flexible substrate using an AJP process.^[^ ^188^ ^]^ The printed RRAM shows forming free, unipolar behavior, lower switching voltage, and operating power than similar flexible RRAM. The printed devices retain their functionality even after bending, making them suitable for monolithically integrated embedded memory in electronic devices (**Figure** **7**a).

Another application is based on WSe_2_ nanosheet networks for electrochemical thin‐film transistors (TFTs).^[^ ^4^ ^]^ In one of the related studies, WSe_2_ nanosheets were exfoliated by liquid‐phase methods in *N*‐methyl 2‐pyrrolidone media. The size selection, along with the solution process and solvent exchange from NMP to isopropanol, provides a further possibility of altering the bandgap and related properties (Figure 7b). For the fabrication, the nanosheet dispersions were sprayed onto flexible alumina‐coated PET substrates to form porous nanosheet networks (PNNs). Such networks appeared uniform over large length scales. However, they show considerable local disorder. In this case, solid electrolytes (polymers or gels) decrease the ionic mobilities, and the use of an ionic liquid/polymer‐based gel increases switching time and speed. In addition, BN nanosheets are sprayed on top of the active layer, not as a dielectric but as an electrochemical separator between the active layer and the conductive top gate.

#### 3.2.4 Molybdenum Diselenide (MoSe_2_)

MoSe_2_ as another member of the TMDs family is important because of its strong optical absorption and high surface activity, thereby exhibiting high efficiency in photoelectrocatalysis. MoSe_2_ is an n‐type semiconductor with an indirect bandgap of 1.09 eV for the bulk and a direct bandgap of 1.57 eV. Possible applications are in the field of batteries and energy storage because of their high surface area and short ionic diffusion length when the number of layers is reduced.^[^ 189, 190, 191 ^]^

A crucial point in 2D materials is attaching different chemical or biological groups from dispersion media. There are few studies related to the functionalization of TMD, which can help to exfoliate them with unsaturated atoms at the edges or defects on the basal plane and vice versa. The functionalization of MoSe_2_ can be categorized into three parts: covalent functionalization, noncovalent functionalization, and metal deposition on the MoSe_2_ sheets.^[^ ^189^ ^]^

In covalent functionalization, the presence of group IV element vacancies (for instance, vacancy of Se in MoSe_2_ structure) and edges plays a crucial role. It is related to the interaction of attaching ligands or any functional groups with unsaturated metal atoms on the edges or defects in the basal plane. The covalent bond formation occurs between free radical or electrophile and Se‐based nucleophile or between organic functional groups and Se vacancies. The organic functional molecules covalently attached to the chalcogen atom drastically change the electronic and optical properties of MoSe_2_.^[^ ^192^ ^]^

**Figure 8:**
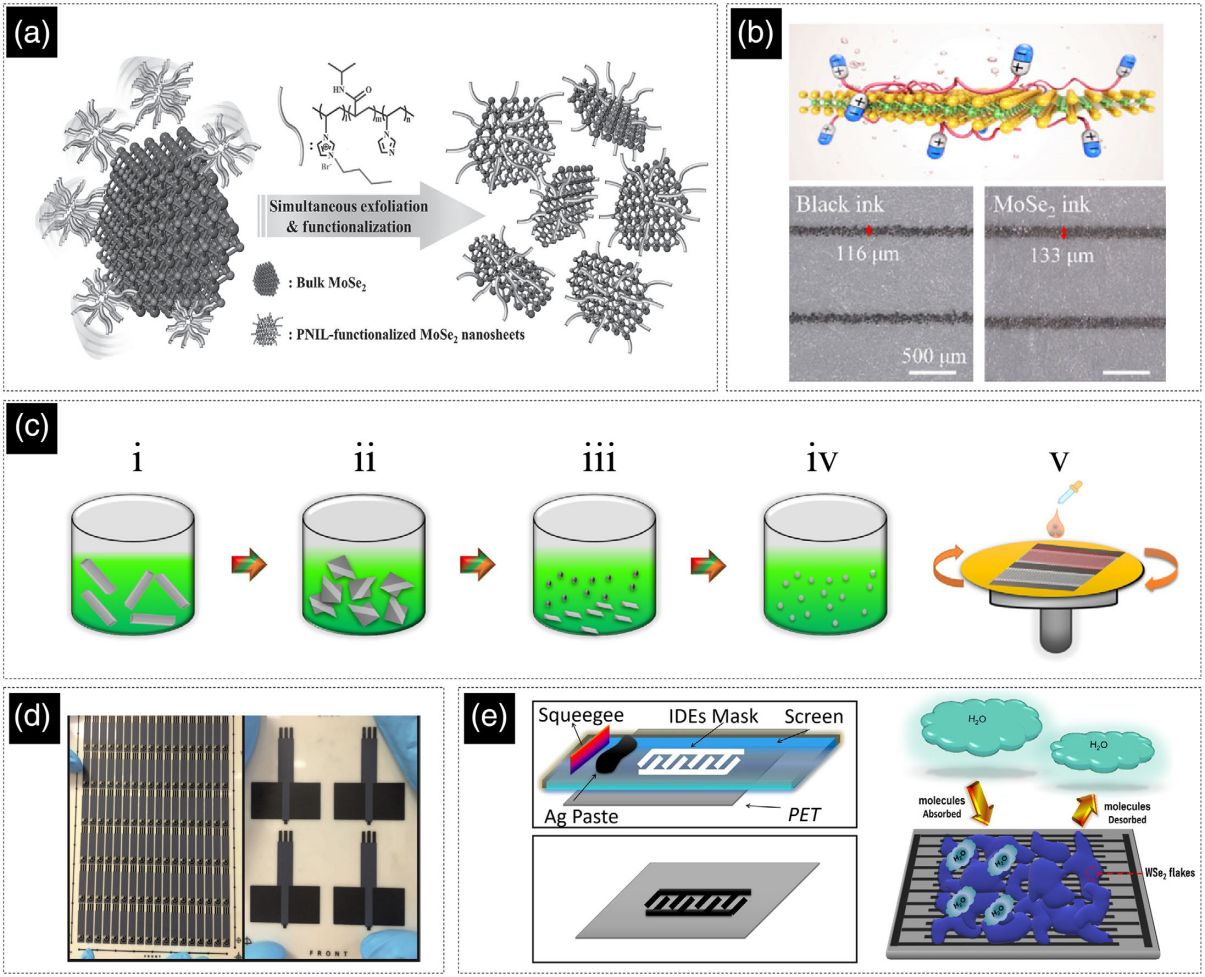
a) Process of simultaneous exfoliating and noncovalently functionalizing MoSe_2_ nanosheets. Reproduced with permission.^[^ ^193^ ^]^ Copyright 2016, Wiley‐VCH. b) A schematic of interaction between TMD nanosheets and zwitterion (top), optical images of commercial black ink and the MoSe_2_ ink printed on A4 paper (bottom). Reproduced with permission.^[^ ^201^ ^]^ Copyright 2020, Tsinghua University Press and Springer‐Verlag GmbH Germany, part of Springer Nature. c) Different steps of MoSe_2_ ink preparation from grounding the bulk material, sonication, and size selection with cascade centrifugations. Reproduced under the terms of the CC‐BY Creative Commons Attribution 4.0 International license (https://creativecommons.org/licenses/by/4.0).^[^ ^202^ ^]^ Copyright 2020, The Authors, published by Springer Nature. d) Photographs of screen‐printed 2D MoSe_2_ – carbon‐based electrodes, Reproduced with permission.^[^ ^203^ ^]^ Copyright 2017, Royal Society of Chemistry. e) Screen printing of Ag ink to form interdigitated electrodes on PET substrate and schematic of the sensing mechanism based on spin‐coated MoSe_2_ nanoflakes. Reproduced under the terms of the CC‐BY Creative Commons Attribution 4.0 International license (https://creativecommons.org/licenses/by/4.0).^[^ ^204^ ^]^ Copyright 2020, The Authors, published by Springer Nature.

Noncovalent functionalization is a low‐cost process for engaging some molecules to alter the surface characteristics without affecting their electrical structure. This interaction can be assisted with van der Waals forces, physisorption, or electrostatic attractions, and generally, it depends on the density of the defects sites. Typical cationic surfactants and polymers can be noncovalently functionalized through electrostatic interaction, which gives them excellent solubility in both organic solvents and aqueous solutions. At the same time, simultaneous exfoliation and functionalization of MoSe_2_ nanosheets are achieved as shown in **Figure** **8**a.^[^ 192, 193 ^]^ Different exfoliation strategies are listed in **Table** **6**.

**Table 6:** Summary of dispersion media and exfoliation condition for MoSe_2_

Dispersion Media	LPE Conditions	Remarks	References
Aqueous mixture of tetrahydrofuran and acetonitrile	Method: Probe sonication	Gas sensing application	[194]
	Initial concentration: 20 mg mL^−1^	Washing with the same solvent mixture and cascade centrifugation	
	Sonication duration: 2 h	Thickness of layers: 2–3.5 nm (<5 layers)	
	Centrifugation: 8000 rpm for 15 min	Lateral dimensions: various centrifugation steps: 0.6–4.2 μm	
Acetonitrile	Method: Probe sonication	Ethanol sensing	[195]
	Initial concentration: 20 mg mL^−1^	Drop casting	
	Sonication duration: 1 h	Lateral size about 600–800 nm	
	Centrifugation: 3000 rpm for 30 min	Thickness was around 2 nm (3 layers, most <10 layers)	
Ethanol/Water mixture	Pretreatment: Ground MoSe_2_ in NMP, for 1 h	Two‐three layer thick MoSe_2_	[196]
	Method: Probe sonication		
	Initial concentration: 10 mg mL^−1^		
	Sonication duration: 1 h		
	Centrifugation: 3000 rpm for 30 min		
Acetone (36 mL):Water (2 mL):Isopropanol (2 mL)	Method: Bath sonication (50 kHz)	Chemiresistive sensor application	[197]
	Initial concentration: 6 mg mL^−1^	4 h sonication shows optimum duration	
	Sonication duration: 2–10 h		
	Centrifugation: 2000 rpm		
IPA	Method: Sonication	Hydrogen evolution reaction	[198]
	Initial concentration: 10 mg mL^−1^	To synthesize of porous MoSe_2_, IPA in the presence of 2.5 vol% of H_2_O_2_ would be a choice	
	Sonication duration: 4–5 h		
	Centrifugation: 3000 rpm for 40 min		
Toluene	Method: Probe sonication	Photodetectors application	[199]
	Initial concentration: 10 mg mL^−1^	Yield: 0.2 mg mL^−1^	
	Sonication duration: 45 min		
	Centrifugation: 1500 rpm for 30 min		
Aqueous solution sodium deoxycholate salt	Method: Sonication (180 W)	Lateral dimensions: less than 1 μm	[200]
	Initial concentration: 10 mg mL^−1^	Thickness of nanosheets is ≈20 nm for WSe_2_ and ≈15	
	Sonication duration: 8 h		
	Centrifugation: 1000 rpm for 10 min		

Zwitterionic components recently gained significant attention due to the high water solubility and contained both cationic and anionic species with a net charge of zero. Efficient liquid exfoliation synthesis of the nanosheets of TMD can be obtained because of the association of alkyl chain moieties with two ionic species to adhere to the surface of nanosheets. The exfoliation yield depends upon the number of alkyl groups of a zwitterion in the dispersion media. The efficient dispersion of a variety of few‐layered TMD nanosheets with lateral dimensions of several hundred nanometers in water‐soluble zwitterions without the need to use additives such as any surfactant or modifier makes it suitable for ink formulation for conventional IJP (Figure 8b). Furthermore, the water‐soluble Zwitterion can also play the role of additives for adjusting the surface tension of the ink to enhance the wettability of the ink on a substrate. Besides photodetector application, biocompatible Zwitterionic‐assisted TMD inks were reported as a good choice for skin‐patchable electronics based on cell (HeLa cells) viability measurements.^[^ ^201^ ^]^

As a showcase, MoSe_2_ is chosen as an active layer for real‐time sensing applications. MoSe_2_ nanoflakes are exfoliated through a wet grinding process. The MoSe_2_ ultrafine powder is first ground in a pestle and mortar using DMF for 8 h (Figure 8c). The obtained gel mixture is dried by heating at 110 °C for 1 h. Lastly, the MoSe_2_ solution is centrifuged. The resultant supernatant solution is then separated via the decantation process. Finally, 7 mg mL^−1^ MoSe_2_ nanosheets in DMF ink are deposited by a spin coater for 30 s at 17 000 rpm and cured at 100 °C. The 2D layer of MoSe_2_ thus achieved has a very high surface‐area‐to‐volume ratio and high surface roughness providing additional Mo and Se edges for hydrogen and oxygen bonding.^[^ ^202^ ^]^

In another example, screen‐printable 2D‐MoSe_2_ electrocatalytic ink at an optimal ratio (10 wt% besides carbon‐based ink) was implemented to produce electrodes/surfaces that exhibit low hydrogen evolution reaction (HER) due to electronegatively charged Se atoms. Dangling bonds at the edge sites have an affinity for binding electropositive H^+^ atoms. Therefore, these sites are responsible for the 2D‐electrochemical activity toward the HER. Moreover, the screen‐printing fabrication approach provides a lower cost and scalable mass production and results in stability improvements over other traditional techniques such as drop‐casting. A series of screen‐printed electrodes is displayed in Figure 8d.^[^ ^203^ ^]^

## 4 Insulator Inks

### 4.1 Hexagonal Boron Nitride (h‐BN)

2D h‐BN, a graphene structural isomorph, was soon synthesized after the discovery of graphene and since then has been used in numerous applications in printed electronics and energy storage. The in‐plane bonds between the boron and the nitrogen atoms are covalent (*σ*), which offers excellent mechanical strength. The absence of π electrons minimizes surface interactions, giving h‐BN unique physical and chemical properties. On the other hand, the electronegativity difference between two atoms causes partially charged sites (B positive, N negative). Selective functionalization can occur for the 2D h‐BN via the lone pair electrons and empty orbitals of the N and B atoms, respectively.^[^ ^205^ ^]^ Various properties can be obtained using different surface functionalizations; for instance, hydroxylation enhances hydrophilicity, fluorination affects photoluminescent emission, edge amination improves surface acid and base reactions, and point defects intensify catalytic activity.^[^ ^206^ ^]^

Unlike other 2D materials, which show metallic or semiconducting characteristics, the bulk form of h‐BN is considered a typical insulator with a bandgap of ≈6 eV due to the free of charge traps and the dangling bonds on its surface.^[^ ^207^ ^]^ Nevertheless, its bandgap can be modified using edge or surface functionalization by efficient modulation or doping with exotic species. A typical example is the 2D h‐BN doped with fluorine groups that act as a semiconductor with a bandgap energy of 3.1 eV (5.8 eV in the undoped state). In addition to doping and functionalization (commonly by carbon or oxygen), the bandgap modulation of 2D h‐BN can be achieved via defect engineering. Altering the local energy of the surface by adjusting its chemical composition would increase ionic conductivity, catalytic activity, and proton exchange rate, which provides an excellent platform for applications in the energy sector.

Extremely high thermal conductivity, chemical and thermal stability, optical transparency in the visible spectrum, and the electrical insulating nature are some other properties that determine h‐BNs widespread use. Since liquid media are preferred for most top‐down approaches, LPE as its subcategory has attracted a lot of attention. Different LPE methods, such as probe sonication, bath ultrasonication, hydrothermal, ball milling, microwave‐assisted exfoliation, and solvothermal techniques, have been developed to synthesize 2D h‐BN with varying surface functional groups, thickness, and size. Generally, the diffusion of the solvent's ions/molecules into adjacent h‐BN layers causes its exfoliation. Accordingly, the extent of liquid interaction with h‐BN layers and, consequently, the yield is dependent on the exfoliating medium. It is reported that a liquid could interact with the h‐BN through polar covalent bonding, Columbic interactions, and Lewis acid–base interactions. In any case, the electronic properties and doping level of the h‐BN layers will be modified by functionalization.^[^ ^205^ ^]^

Parameters affecting exfoliation or synthesis mainly follow from the significance of surface functionalization or level of doping in energy applications. According to the literature, modification of 2D h‐BN is obtained by functionalizing with long‐chain molecules, amine group [‐NH_2_], and hydroxyl groups [‐OH], doping with oxygen, fluorine, and carbon. Due to the partly ionic character of the interlayer bonds, choosing the proper methods and conditions among several exfoliation procedures is essential in the production process for printed electronic inks.^[^ ^205^ ^]^ Some of the exfoliation conditions and results are listed in **Table** **7**. However, not all of the methods can be applied for exfoliating and using them as a solution‐based technique for ink formulation at the same time. Another parameter that should be focused on is the rheological properties of the inks that must be fitted to the printing or coating process's viscosity ranges.

**Table 7:** Summary of dispersion media and exfoliation condition for h‐BN

Dispersion media	LPE conditions	Remarks	References
1,2‐dichloroethane solutiona)	Method: Sonication	<4 Layers (few layers)	[208]
	Initial concentration: 0.04 mg mL^−1^		
	Sonication duration: 1 h		
	Centrifugation: ‐		
Methanesulfonic acid and NMP	Method: Bath sonication	Yield: 0.3 mg mL^−1^	[209]
	Initial concentration: 2 mg mL^−1^		
	Sonication duration: 8 h		
	Centrifugation: ‐	Flake layers: <10 layers (less than 3 nm)	
Acetone, methanol, ethanol, 1‐propanol, 2‐propanol, and tert‐butanol in water	Method: Bath sonication	Most effective media: 60 wt% of tert‐butanol in Water	[210]
	Initial concentration: 2 mg mL^−1^		
	Sonication duration: 3 h	Few‐layered sheets (≈7–9 nm)	
	Centrifugation: 3200 rpm for 20 min		
Ethanol and water	Method: Sonication	Yield: 0.075 mg mL^−1^	[211]
	Initial concentration: 3 mg mL^−1^	Few‐layer nanosheets (thickness of 3–4 nm)	
	Sonication duration: 8 h		
	Centrifugation: 3000 rpm for 20 min		
DMF	Method: Tip sonication	Yield: 0.5–1 mg mL^−1^	[212]
	Initial concentration: 25 mg mL^−1^	Thickness of layers are less than 7 nm	
	Sonication duration: 10 h		
	Centrifugation: 5000–8000 rpm		
Water	Method: Bath sonication (40 kHz)	Yield: 0.05 mg mL^−1^	[213]
	Initial concentration: 2 mg mL^−1^	Thickness of products are less than 10 nm (<30 Layers)	
	Sonication duration: 8 h		
	Centrifugation: 3000 g		
Amonia:IPA (3:2)	Method: Bath sonication (40 kHz)	Yield: 0.022 mg mL^−1^	[214]
	Initial concentration: 20 mg mL^−1^		
	Sonication duration: 35 h		
	Centrifugation: 3000 g		
Thionyl chloride	Method: Bath sonication (40 kHz)	Yield: 0.37–0.40 mg mL^−1^	[207]
	Initial concentration: 2 mg mL^−1^		
	Sonication duration: 20 h		
	Centrifugation: 2000–2500 rpm for 5 min		

a) Poly(*m*‐phenylenevinylene*‐co*‐2,5‐dictoxy‐*p*‐phenylenevinylene).

**Figure 9:**
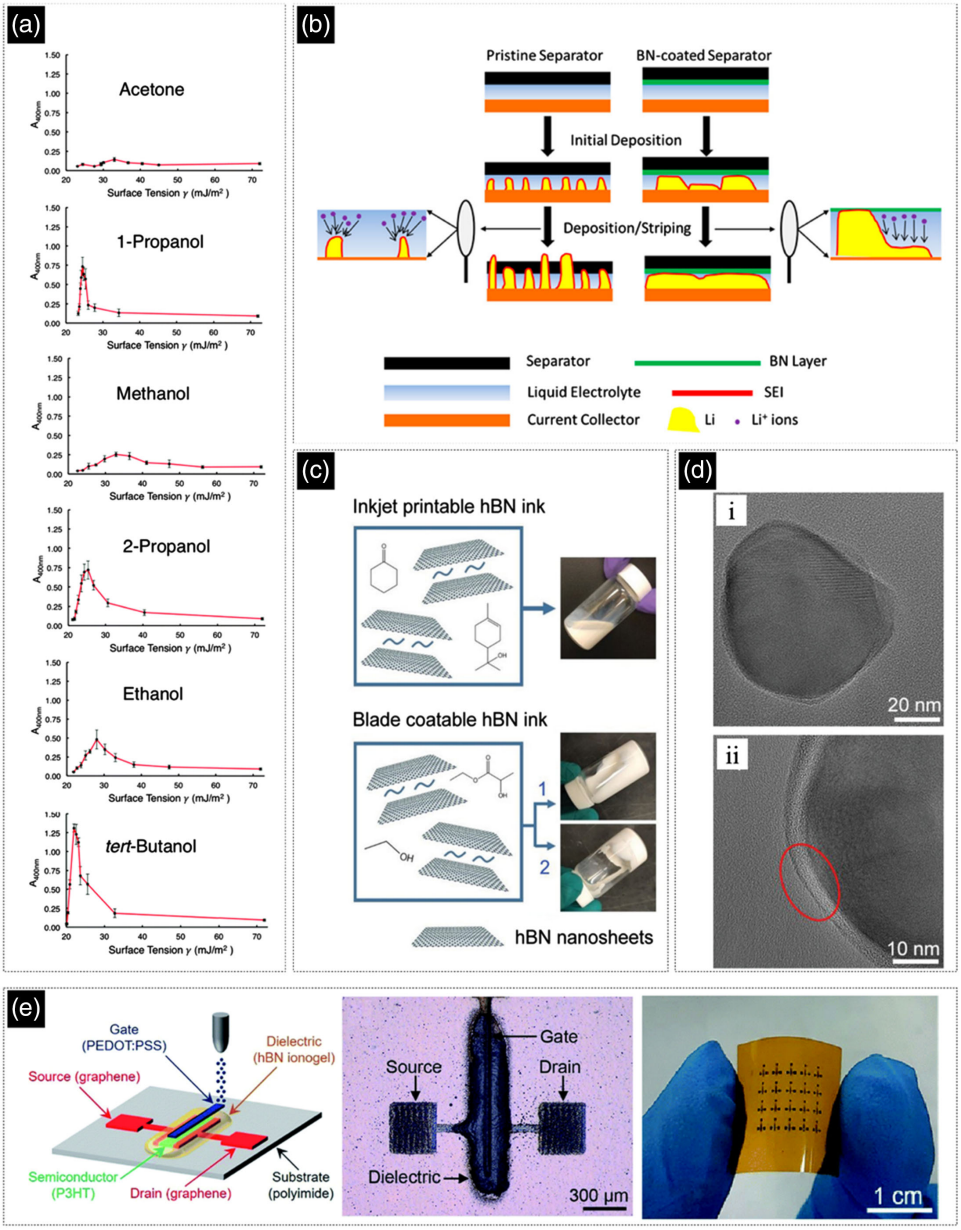
a) UV–vis data for h‐BN nanosheets in different co‐solvent systems recorded at 400 nm vs. surface tension. Each case represents different ratios between solvent and water w/w%. Reproduced with permission.^[^ ^210^ ^]^ Copyright 2015, Royal Society of Chemistry. b) Effect of a boron nitride nanosheet‐coated separator for Li metal anode. In a typical separator (right), Li dendrites are grown on the current collector during Li deposition, resulting in low Coulombic efficiency; however, a thermally conductive BN coating can result in a uniform deposition/stripping of Li due to the smaller total surface area of the initial deposited Li wires and decreasing the risk of dendritic Li growth and crack cause improving performance. Reproduced with permission.^[^ ^216^ ^]^ Copyright 2015 American Chemical Society. c) h‐BN ink formulation for the IJP is prepared by dispersion of the h‐BN/EC exfoliated powder in an 85:15 ratio of cyclohexanone and terpineol, and for the blade coating, h‐BN inks are prepared by dispersing h‐BN/EC powder in a 2:1 ratio of ethanol and ethyl lactate where a more viscous ink is required than IJP method. d) Morphology of exfoliated h‐BN nanosheets by shear mixing. TEM images showing the EC coating on the surface of the nanosheets at lower (i) and higher (ii) magnification, Reproduced with permission.^[^ ^217^ ^]^ Copyright 2019, Wiley‐VCH. e) Schematic, optical microscopy, and photograph image of a fully printed thin‐film transistor (TFT) with the h‐BN ionogel dielectric on a polyimide film. Reproduced with permission.^[^ ^218^ ^]^ Copyright 2021, Royal Society of Chemistry.

As mentioned, boron nitride can be exfoliated in a wide range of dispersion media, including aqueous and organic systems (**Figure** **9**a).^[^ ^210^ ^]^ One of the solvents that has been reported for effective exfoliation of h‐BN is DMF. This is due to the polar nature of the DMF, along with the assistance of the cavitation phenomena in the continuous sonication procedure. The exfoliation yield can be increased by using polymeric materials such as polycarbonate (PC), polyvinyl butyral, and poly(methyl methacrylate). Physical interactions of polymer chains with the surface of exfoliated nanoflakes help separate the layers. Furthermore, this interaction increases the stability of the dispersion because of the steric hindrance and repulsion mechanism between nanoflakes connected with polymer. PC, one of the materials mentioned, has a high glass‐transition temperature (≈150 °C) and high solubility at room temperature in the exfoliation solvent media such as DMF, which is well fitted to formulate suitable inks. For methods demanding higher viscosity like screen printing (>10 Pa s at 10 s^−1^ shear rate), a low‐boiling‐point solvent such as chloroform can be implemented as a viscosity tuning agent and control the drying process, which also affects the adhesion of the ink to the substrates.^[^ ^215^ ^]^

2D h‐BN plays an important role as an electrode separator in Li‐ion batteries (LIBs).^[^ ^217^ ^]^ Separators are of utmost importance in preventing short circuits between the battery electrodes as well as controlling ion transfer during the charging and discharging processes. Short circuits are a major concern for practicality and safety concerns in LIBs. This problem can be avoided by the utilization of a separator with high thermal stability. In addition, it has been reported that the presence of h‐BN layers can suppress Li‐dendrite formation during the charging process. Dendrites eventually can penetrate the separator, also leading to a short circuit (Figure 9b).^[^ ^216^ ^]^

Another interesting investigation in the ink formulation field was conducted to synthesize and stabilize h‐BN. EC‐ethanol solution was used as the exfoliation medium, promoting the synthesis and stabilization of h‐BN. Furthermore, EC not only minimizes the tendency of nanoflakes to agglomerate but also enables control over the ink rheology for a wide range of ink viscosities, allowing ink formulations from low‐viscosity printing methods (e.g., IJP and spray coating) to high‐viscosity techniques (e.g., screen printing and blade coating). For instance, for IJP, cyclohexanone and terpineol solvents were added to h‐BN and tuning the viscosity to 8.0 × 10^−3^ Pa s at 1000 s^−1^. For blade coating (Figure 9c), a mixture of ethanol and ethyl lactate (2:1) as a lower boiling point solvent system was considered (0.4 Pa s at 1000 s^−1^) to even provide an ink with higher viscosity by partial evaporation of the solvents (2.6 Pa s at 1000 s^−1^). In the heat treatment step, the printed patterns were annealed at 300 °C for 30 min; the decomposition of EC polymer causes a high porosity film (Figure 9d). This carbonaceous film on h‐BN nanoflakes improves the organic phase wettability, which is an essential property for battery separators in contact with electrolytes.^[^ ^217^ ^]^

Another application of h‐BN in the battery field is as a matrix material for solid‐state electrolytes. h‐BN amends the performance of electrolytes by improving the fabrication robustness and mechanical stability and also increments of the Li transference number (Li^+^) and ionic conductivity. Still, the presence of inorganic fillers suppresses ion transport affecting the cell capacity of the battery. On the contrary, the smaller size of functionalized 2D h‐BN layers are capable of both boosting the electrolyte in terms of ionic conductivity and mechanical strength and dealing with such issues.^[^ ^205^ ^]^

Another asset of the 2D h‐BN in this context is the ability to formulate ionogel printable inks. Exfoliation and ink formulation of h‐BN with residual chemicals does not cause adverse effects for dielectric applications. The organic materials enter the ink system via the exfoliation process, and the decomposition of these materials in the heating process leaves an amorphous carbon thin film on the surface of h‐BN, which improves the interactions between ionic liquids and h‐BN nanoplates. For ink formulation, nanoplatelets are mixed with 1‐ethyl‐3‐methylimidazoliumbis (trifluoromethylsulfonyl) imide (EMIM‐TFSI) and ethyl lactate as an ionic liquid and solvent, respectively, with the ratio of 1:2 between solid content and ionic liquid. These ion gels contain a storage modulus higher than its loss modulus over the entire measured frequency range. The mechanical strength and ion conductivity can be varied with h‐BN concentration. The ion gels of h‐BN were printed by the AJP method on a polyimide film as dielectric for fully printed thin‐film transistor applications (see Figure 9e).^[^ ^218^ ^]^

## 5 Perspective

For graphene inks, as a result of vast research efforts, well‐performing exfoliation methods and ink formulations could be established. The situation is less clear for the other 2D materials hexagonal boron nitride (h‐BN), the transition metal dichalcogenides (TMDs), and black phosphorous (BP). The very different attempts to formulate suspensions and inks for printing are an indication that research here is in a rather early stage. It can be envisioned though that these materials will play a leading role in the design of future electronic devices. We gave a detailed overview of the current state of research and challenges concerning exfoliation, ink formulation, and applications of these materials, hoping to help overcome reservations that may exist toward experimenting with 2D materials other than graphene‐related compounds.

## Conflict of Interest

The authors declare no conflict of interest.
